# Global burden and health inequality of atrial fibrillation/atrial flutter from 1990 to 2021

**DOI:** 10.3389/fcvm.2025.1585980

**Published:** 2025-05-21

**Authors:** Xia Li, Zhen Li, Hongtao He, Sizhou Wang, Honglei Su, Guobin Kang

**Affiliations:** ^1^ Department of Cardiology 1, Hebei Provincial Hospital of Chinese Medicine, Shijiazhuang, Hebei, China; ^2^ Department of Child Healthcare, The Fourth Hospital of Shijiazhuang, Gynecology and Obstetrics Hospital Affiliated to Hebei Medical University, Shijiazhuang, Hebei, China; ^3^ Internal Medicine 1, Xinle Hospital of Traditional Chinese Medicine, Shijiazhuang, Hebei, China

**Keywords:** atrial fibrillation, atrial flutter, global burden of diseases, health inequality, predictions

## Abstract

**Introduction:**

Atrial fibrillation (AF) and atrial flutter (AFL) are the most prevalent tachyarrhythmias worldwide, leading to severe complications such as stroke and heart failure. Despite advancements in diagnosis and management, the global burden of AF/AFL continues to increase, highlighting the urgent need for comprehensive epidemiological research.

**Methods:**

This study analyzed data from the Global Burden of Disease (GBD) 2021 to examine the incidence, prevalence, mortality, and disability-adjusted life years (DALYs) associated with AF/AFL from 1990 to 2021. Age-standardized rates were calculated at global, regional, and country-specific levels. Decomposition and Bayesian age-period-cohort models were used to explore trends and forecast future disease burdens.

**Results:**

In 2021, AF/AFL contributed to 4.48 million new cases globally, with an age-standardized incidence rate (ASIR) of 52.1 per 100,000. The global prevalence reached 52.55 million cases, while the disease caused 338,947 deaths. The global age-standardized DALY rate stood at 101.4 per 100,000. Significant disparities were observed, with higher disease burdens in high Socio-demographic Index (SDI) regions compared to low SDI regions. Between 1990 and 2021, aging and population growth were key drivers of the increased burden, with regional variations associated with economic development and healthcare access. Bayesian projections indicate a gradual rise in incidence and prevalence through 2050, although mortality and DALY rates are expected to decline.

**Conclusions:**

AF/AFL presents a substantial public health challenge, with rising incidence and prevalence primarily driven by demographic changes and enhanced diagnostic capabilities. Addressing health inequalities requires targeted interventions and strengthening healthcare systems. Future strategies should prioritize prevention and equitable access to advanced therapies.

## Introduction

Atrial fibrillation (AF) and atrial flutter (AFL) are the most prevalent tachyarrhythmias encountered in clinical practice, often leading to severe complications such as myocardial infarction, heart failure, and stroke. These complications significantly impact patients' physical and mental well-being, ultimately reducing their quality of life (1, 2). The global prevalence of AF/AFL has doubled from 1990 to 2019, reaching 59.7 million cases, underscoring its widespread occurrence. This upward trajectory is expected to continue, accompanied by an increase in the absolute number of disability-adjusted life years (DALYs) attributed to AF/AFL, which reached 5.97 million and emerged as a leading cause of disability worldwide (3).

The economic burden of AF/AFL is substantial, with high medical expenditures per patient. In the United States, direct costs range from US$2,000 to US$14,200 annually, while in Europe, expenses vary between €450 and €3,000 per patient per year (4). Moreover, hospitalizations due to AF have surged in recent years, placing an increasing strain on both patients and healthcare systems globally (5).

The pathogenesis of AF/AFL is multifactorial, with primary risk factors including hypertension, smoking, alcohol consumption, high sodium intake, and obesity. Additionally, sociodemographic variables such as population size, age distribution, and racial composition, alongside socioeconomic factors like income levels, education, and healthcare accessibility, play crucial roles in shaping the burden of AF/AFL (6–8). Prior studies have demonstrated that in underdeveloped regions, limited education and low literacy levels are associated with a heightened disease burden (9, 10). Conversely, in high-income countries with greater access to medical resources and higher educational attainment, the incidence of AF/AFL remains elevated. However, the availability of advanced diagnostic and therapeutic measures has contributed to improved survival rates (11). The rising global burden of AF/AFL may be attributed to enhanced detection capabilities, an increasing prevalence of risk factors, and prolonged life expectancy due to medical advancements (12).

Despite the well-documented regional disparities and international inequalities in AF/AFL incidence, current studies lack comprehensive epidemiological data encompassing all geographical regions. Leveraging data from the Global Burden of Disease (GBD) database can facilitate a more nuanced understanding of AF/AFL burden across different countries and regions. Additionally, big data analysis enables the identification of variations in AF/AFL incidence across gender and age groups (13). The Bayesian age-period-cohort (BAPC) model offers a dynamic approach to predicting future trends in AF/AFL disease burden (14).

In this study, we present the most recent statistics on AF/AFL across 204 countries and regions from 1990 to 2021, examining incidence, prevalence, mortality, DALYs, and health inequality indicators. Our findings aim to provide comprehensive insights to support the global prioritization and allocation of healthcare resources, with the ultimate goal of addressing health disparities and mitigating the disease burden associated with AF/AFL.

## Methods

### Overview

The Global Burden of Disease (GBD) 2021 study offers comprehensive epidemiological estimates for 371 diseases and injuries by age and sex across 204 countries and territories, providing annual data from 1990 to 2021 at global, regional, and national levels. Estimates of AF/AFL (ICD-10: I48–I48.9) burden were extracted from the publicly accessible GBD Results Tool (https://vizhub.healthdata.org/ gbd-results/) (15). A key metric used in the study is the Sociodemographic Index (SDI), which ranges from 0 to 1 and summarizes social and economic conditions related to health outcomes in each location. The SDI considers variables such as fertility rates among individuals under 25, educational attainment for those aged 15 and above, and per capita income, all of which are closely associated with health outcomes. Based on their SDIs, the 204 countries and territories included in the study are categorized into five sociodemographic quintiles: low, low-middle, middle, high-middle, and high (16).

### Data sources

For the GBD 2021 study, data were collected through systematic assessments of surveys, censuses, civil registrations, demographic surveillance, and other health-related sources. The risk of bias for each data source was evaluated and adjusted using standardized statistical estimation with DisMod-MR 2.1, a Bayesian meta-regression tool (15). This study specifically evaluated the incidence, prevalence, mortality, and DALY burden associated with Atrial Fibrillation and Flutter (AF/AFL), adhering to the GBD protocol and the Guidelines for Accurate and Transparent Health Estimates Reporting (GATHER).

### Burden description

This investigation provides comprehensive annual epidemiological metrics encompassing incidence, prevalence, mortality, and DALYs to systematically quantify the disease burden of AF/AFL. All outcomes are expressed as crude values and corresponding age-standardized rates (ASR) per 100,000 population, accompanied by 95% uncertainty intervals (UI) derived from 1,000 stochastic computational iterations, representing the 2.5th to 97.5th percentile distributions. The age-standardized parameters—including incidence rate (ASIR), prevalence rate (ASPR), mortality rate (ASMR), and DALY rate (ASDR)-were computed utilizing the direct standardization methodology referenced in the Global Burden of Disease analytical framework, enabling cross-population comparability by adjusting for demographic heterogeneity in age structure and population size. The DALY metric constitutes a composite health gap measure integrating both mortality and morbidity components, capturing the cumulative loss of health expectancy attributable to AF/AFL through premature mortality and non-fatal disability weights. The standardization process adheres to the formula:

$$ASR=\sum_{i=1}^{n}(\frac{Ri\times P_{i}^{std}}{P_{i}})$$

Where R_i_ is the disease incidence or mortality (i.e., age rate), P_i_ is the population in the studied population, P_i_^std^ is the population proportion of the age group in the standard population, and *n* is the number of age group.

### Joinpoint regression

The joinpoint regression model is a collection of linear statistical models used to analyze trends in disease burdens attributable to Atrial Fibrillation and Flutter (AF/AFL) over time. This model estimates changes in disease rates using the least squares method, which avoids the biases typically associated with trend analyses based solely on linear assumptions. The turning point of the shifting trend is determined by calculating the sum of squared residual errors between the estimated and actual values. The Joinpoint software (version 4.9.1.0; National Cancer Institute, Rockville, MD, USA) was employed to construct this model (17).

To quantitatively describe the trends in the onset, prevalence, mortality, and DALYs of AF/AFL, we used the Average Annual Percentage Change (AAPC) and Annual Percentage Change (APC) metrics. These were applied across the years 2021 globally, across 5 SDI regions, 21 GBD regions, and 204 countries. The AAPC is accompanied by its 95% confidence interval (CI) to reflect the uncertainty in response estimation. If the 95% CI of the AAPC estimate is greater than 0, the age-standardized indicator indicates an increasing trend; if less than 0, it indicates a decreasing trend; and if the CI includes 0, the trend is stable (18).

### Decomposition analysis

A decomposition methodology was used to determine the contributions of aging, population growth, and epidemiological changes to the incidence, prevalence, DALYs, and mortality of AF/AFL (19). This analysis helps to break down the underlying factors influencing the observed disease burden trends over time. The DALYs at each specific location were derived using the following formula:

$$DALY_{ax,px,ex}=\sum_{i=1}^{20}\left(a_{i,x}\times \;p_{x}\times \;e_{i,x}\right)$$

where DALY a_x_, p_x_, e_x_ represented DALYs computed based on the dynamics of aging, population growth, and DALYs rate for year x. The a_i,x_ denoted the fraction of the population within age group i across the 20 age group in year x. The p_x_ signified the total population in year x. The e_i,x_ indicated DALYs rate for age group i in year x. The influence of each factor on the shift in DALYs from 1990 to 2021 was determined by assessing the impact of altering one factor while keeping the others constant.

### Health inequality

To assess health inequality, we used the Slope Index of Inequality (SII), an absolute measure of inequality. SII was calculated by regressing the DALY rate on the relative social status scale associated with the SDI, which was defined by the midpoint of the population cumulative category interval sorted by SDI. In this study, SII represents the estimated difference in DALY rates between countries or regions with the lowest and highest SDI while considering population size using an appropriate regression model.

To calculate SII, the entire population was ranked from the lowest to highest SDI in a weighted manner, accounting for the proportional distribution of the population within each subgroup. A greater absolute value of SII indicates a higher degree of health inequality (20).

### Bayesian age-period-cohort (BAPC) analysis

To predict the ASR from 2022 to 2050 by sex, we employed a Bayesian Age-Period-Cohort (BAPC) analysis using the BAPC and INLA packages in R. The BAPC model relies on an integrated nested Laplacian approximation, which approximates marginal posterior distributions and avoids mixing and convergence issues that often arise with the Markov Chain Monte Carlo sampling technique traditionally used in Bayesian methods (21).

All statistical analyses and visualizations in this study were performed using R (version 4.2.2). Differences were considered statistically significant at two-sided *P* < 0.05.

## Results

### Global burden of AF/AFL in 2021

In 2021, Atrial Fibrillation and Flutter (AF/AFL) contributed significantly to the global health burden, with a total of 4,484,926 new cases recorded worldwide. The global age-standardized incidence rate (ASIR) was 52.12 per 100,000 people. Additionally, the prevalence of AF/AFL reached 52,552,045 cases, with an age-standardized prevalence rate (ASPR) of 620.51 per 100,000.

**Figure 1:**
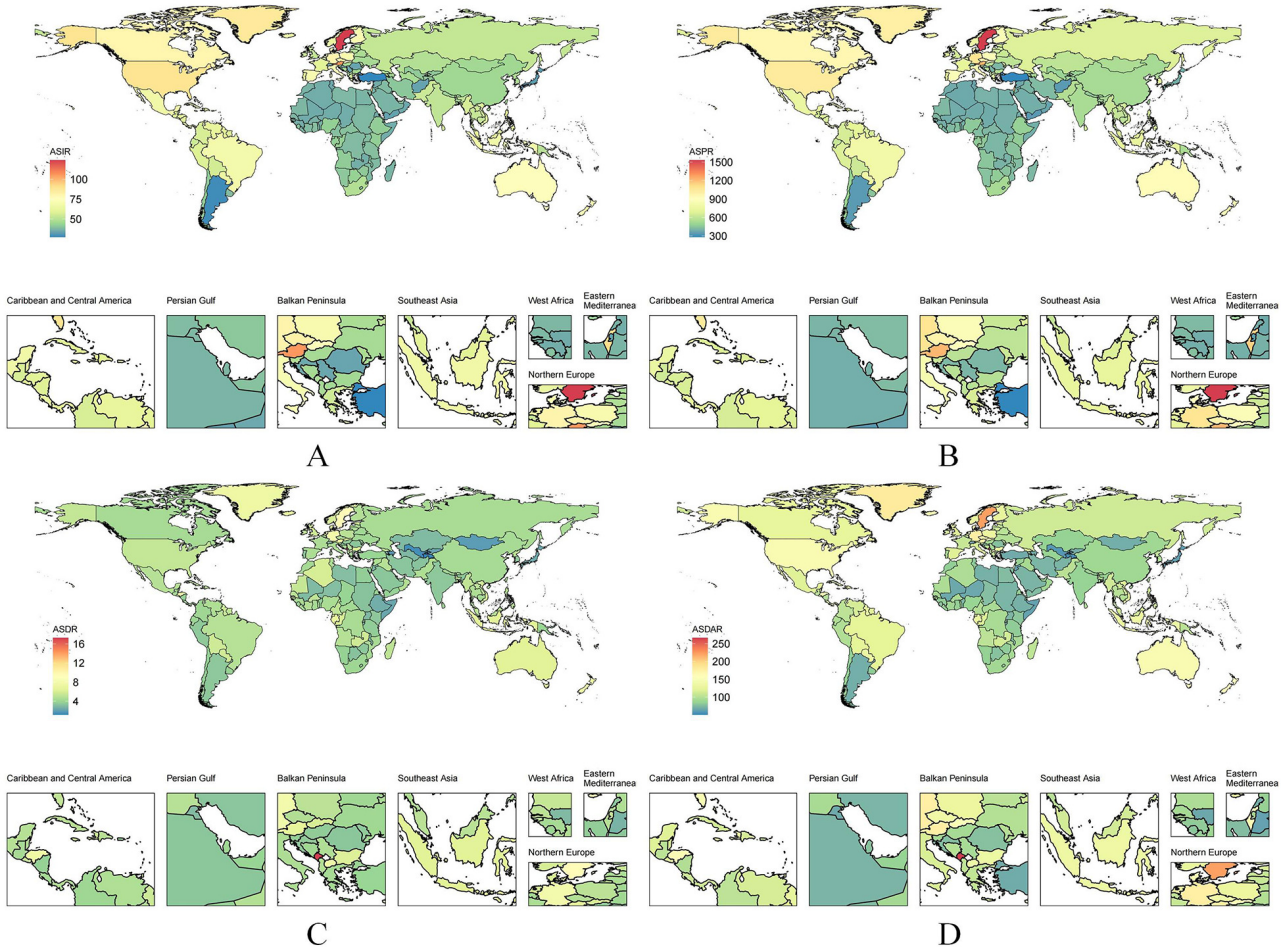
The ASR of AF/AFL among all countries/territories in 2021. **(A)** ASIR; **(B)** ASPR; **(C)** ASMR; **(D)** ASDR.

Mortality associated with AF/AFL resulted in 338,947 deaths, corresponding to an age-standardized mortality rate (ASMR) of 4.36 per 100,000. The global disability-adjusted life years (DALYs) caused by AF/AFL totaled 8,358,894, with an age-standardized DALYs rate (ASDR) of 101.4 per 100,000 (Supplementary Table S1; Figures 1A–D).

### Burden across different socio-demographic Index (SDI) regions

The burden of atrial fibrillation and atrial flutter (AF/AFL) exhibited substantial variation across different Socio-Demographic Index (SDI) regions. High SDI regions experienced the greatest burden of the disease. In 2021, these regions recorded 1,334,730 new AF/AFL cases, with an age-standardized incidence rate (ASIR) of 65.1 per 100,000 people. The prevalence in these regions was 17,401,068 cases, corresponding to an age-standardized prevalence rate (ASPR) of 788.35 per 100,000. Additionally, high SDI regions reported 125,622 deaths [age-standardized mortality rate (ASMR): 4.66 per 100,000] and a total of 2,788,952 disability-adjusted life years (DALYs), with an age-standardized DALY rate (ASDR) of 118.88 per 100,000 (Supplementary Table S1).

Similarly, middle SDI regions also bore a considerable AF/AFL burden, with 1,330,376 new cases reported in 2021, corresponding to an ASIR of 51.11 per 100,000. The prevalence in these regions reached 14,670,503 cases (ASPR: 579.06 per 100,000), while the total number of deaths stood at 83,853, with an ASMR of 4.26 per 100,000. The overall DALY burden in middle SDI regions was 2,256,889, with an ASDR of 96.28 per 100,000 (Supplementary Table S1).

In contrast, low SDI regions exhibited a significantly lower AF/AFL burden. In 2021, these regions recorded only 198,494 new cases, with an ASIR of 43.25 per 100,000. The prevalence was 1,959,344 cases (ASPR: 463.23 per 100,000), while the number of deaths was 10,623 (ASMR: 3.74 per 100,000). The total DALY burden in low SDI regions was 321,963, with an ASDR of 84.52 per 100,000 (Supplementary Table S1).

### Burden in specific countries and regions

The burden of AF/AFL was particularly pronounced in certain regions. For instance, East Asia reported 953,898 new cases in 2021, with an ASIR of 45.11 per 100,000. The prevalence in this region reached 11,215,165 cases (ASPR: 526.44 per 100,000), while the number of deaths was 67,666 (ASMR: 4.3 per 100,000). The total DALY burden in East Asia amounted to 1,723,468, with an ASDR of 89.83 per 100,000 (Supplementary Table S1; Figures 1A–D).

South Asia also experienced a substantial AF/AFL burden, with 698,920 new cases reported in 2021, corresponding to an ASIR of 51.01 per 100,000. The prevalence in this region was 6,882,583 cases (ASPR: 530.9 per 100,000), while the total number of deaths was 36,165 (ASMR: 3.69 per 100,000). The overall DALY burden in South Asia amounted to 1,071,260, with an ASDR of 88.3 per 100,000 (Supplementary Table S1; Figures 1A–D).

Western Europe exhibited a notably high AF/AFL burden, with 600,735 new cases recorded in 2021. The ASIR in this region was 68.19 per 100,000, while the prevalence reached 8,410,140 cases (ASPR: 844.93 per 100,000). The total number of deaths was 72,184 (ASMR: 5.52 per 100,000), and the DALY burden amounted to 1,440,284, with an ASDR of 131.15 per 100,000, reflecting a particularly high disease burden (Supplementary Table S1; Figures 1A–D).

Conversely, regions with lower AF/AFL incidence, such as Central Sub-Saharan Africa, reported considerably smaller burdens. In 2021, this region recorded only 19,101 new cases, with an ASIR of 39.56 per 100,000. The prevalence was 182,594 cases (ASPR: 424.57 per 100,000), while the total number of deaths was 1,311 (ASMR: 4.67 per 100,000). The overall DALY burden in Central Sub-Saharan Africa was 37,999, with an ASDR of 98.45 per 100,000.

Similarly, Oceania reported 3,484 new AF/AFL cases in 2021, with an ASIR of 52.33 per 100,000. The prevalence in this region was 34,994 cases (ASPR: 578.44 per 100,000), while the total number of deaths stood at 185 (ASMR: 4.29 per 100,000). The overall DALY burden in Oceania was 6,606, with an ASDR of 110.15 per 100,000 (Supplementary Table S1; Figures 1A–D).

### Trends from 1990 to 2021

#### Sex differences in AF/AFL burden

**Figure 2:**
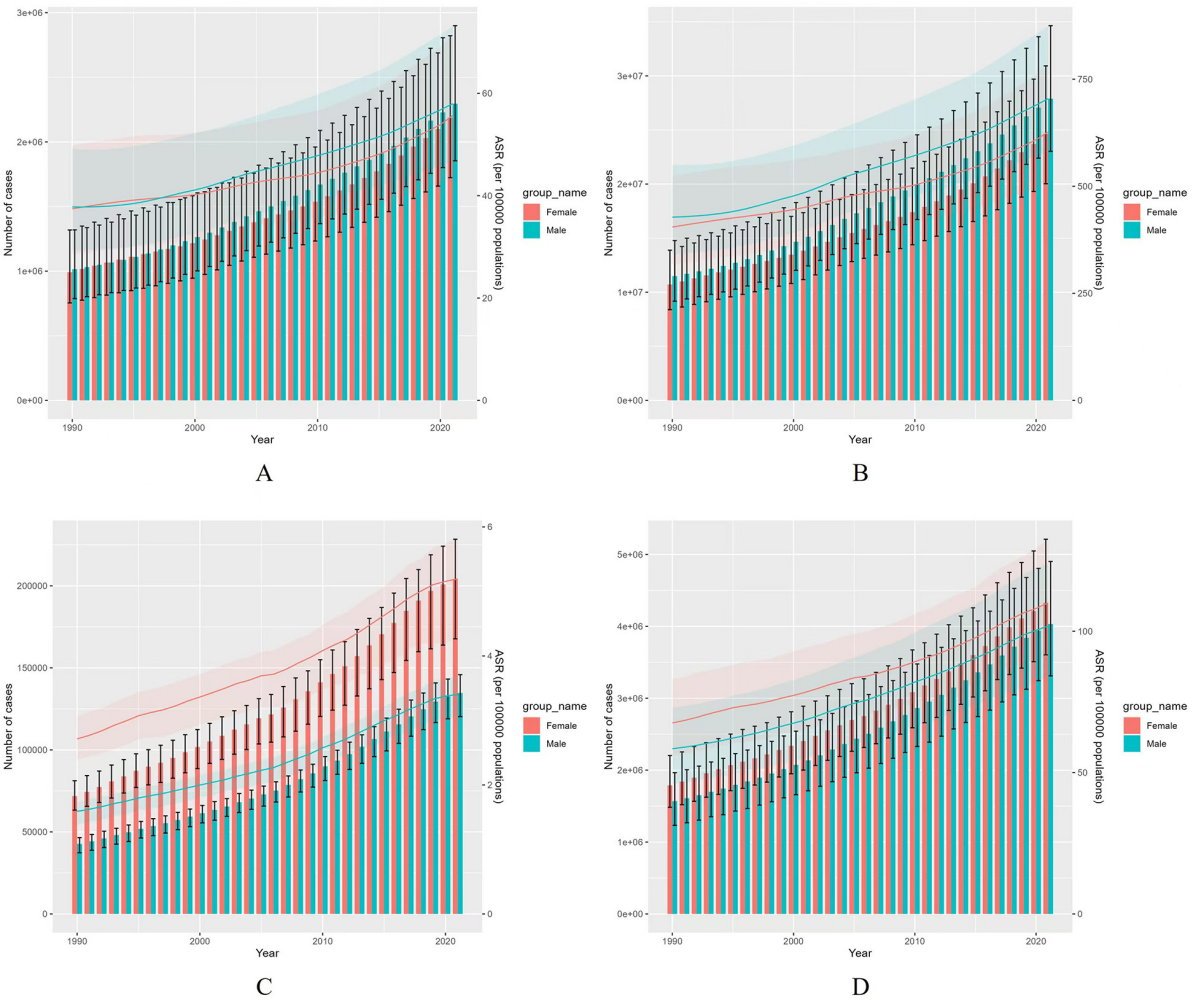
Comparison of incidence, prevalence, mortality, and DALYs rates between males and females globally from 1990 to 2021 based on both full-age cases and age-standardized measures. **(A)** Incident cases and ASIR; **(B)** Prevalent cases and ASPR; **(C)** Death cases and ASMR; **(D)** DALYs counts and ASDR. Bar charts represent counts; lines represent age-standardized rates.

Globally, the burden of AF/AFL was slightly higher in males than females, with relatively stable male-to-female ratio patterns observed from 1990 to 2021. Both sexes exhibited similar trends in the overall increase in incidence over the past 32 years. The age-standardized incidence rate (ASIR) for AF/AFL showed a gradual upward trend for both males and females (Figure 2A). Similarly, the prevalence and age-standardized prevalence rate (ASPR) for AF/AFL demonstrated a clear increase from 1990 to 2021 (Figure 2B).

In terms of deaths and their respective age-standardized mortality rates (ASMRs) for AF/AFL, an overall increasing trend was observed for both sexes from 1990 to 2021. However, females exhibited significantly higher rates than males (Figure 2C). Additionally, disability-adjusted life years (DALYs) and their corresponding age-standardized DALY rates (ASDRs) showed a clear upward trend for both sexes, with females consistently having higher rates than males (Figure 2D).

### Age differences in AF/AFL burden

**Figure 3:**
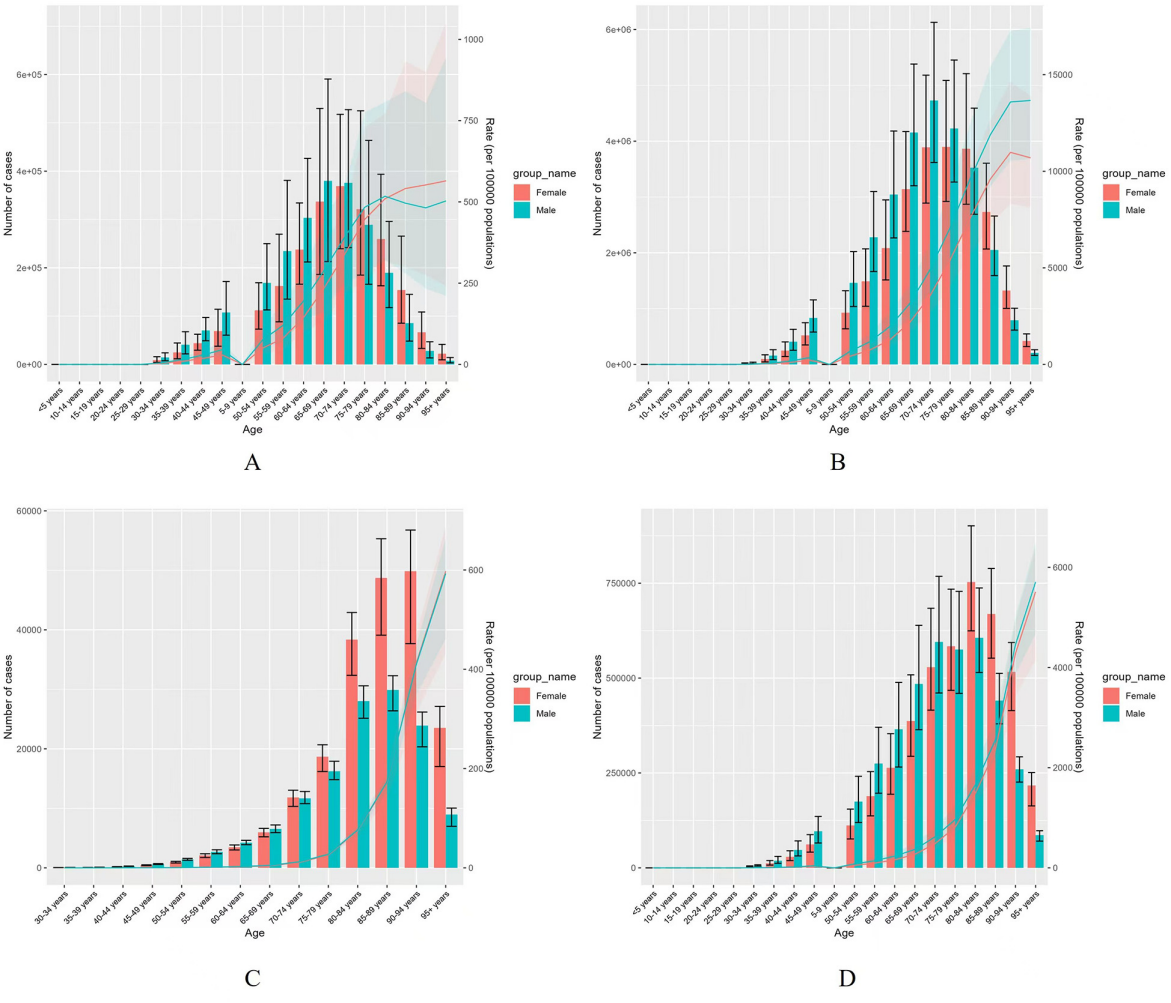
Comparison of the global incidence, prevalence, mortality rates, and DALYs counts among males and females by age group in 2021. **(A)** Incident cases and CIR; **(B)** Prevalent cases and CPR; **(C)** Death cases and CMR; **(D)** DALYs counts and CDR; Bar charts represent counts; lines represent crude rates.

The global burden of AF/AFL was markedly higher in the age groups 60–64 years, 65–69 years, 70–74 years, and 75–79 years, indicating an increase in the burden of the disease with age in 2021 (Figures 3A–D). Deaths due to AF/AFL were most pronounced in the age groups 80–84 years, 85–89 years, and 90–94 years, with females exhibiting significantly higher death rates than males (Figure 3C). Additionally, both crude mortality rates and DALY rates increased exponentially with age for both sexes (Figures 3C,D).

### Fluctuations in global burden from 1990 to 2021

**Figure 4:**
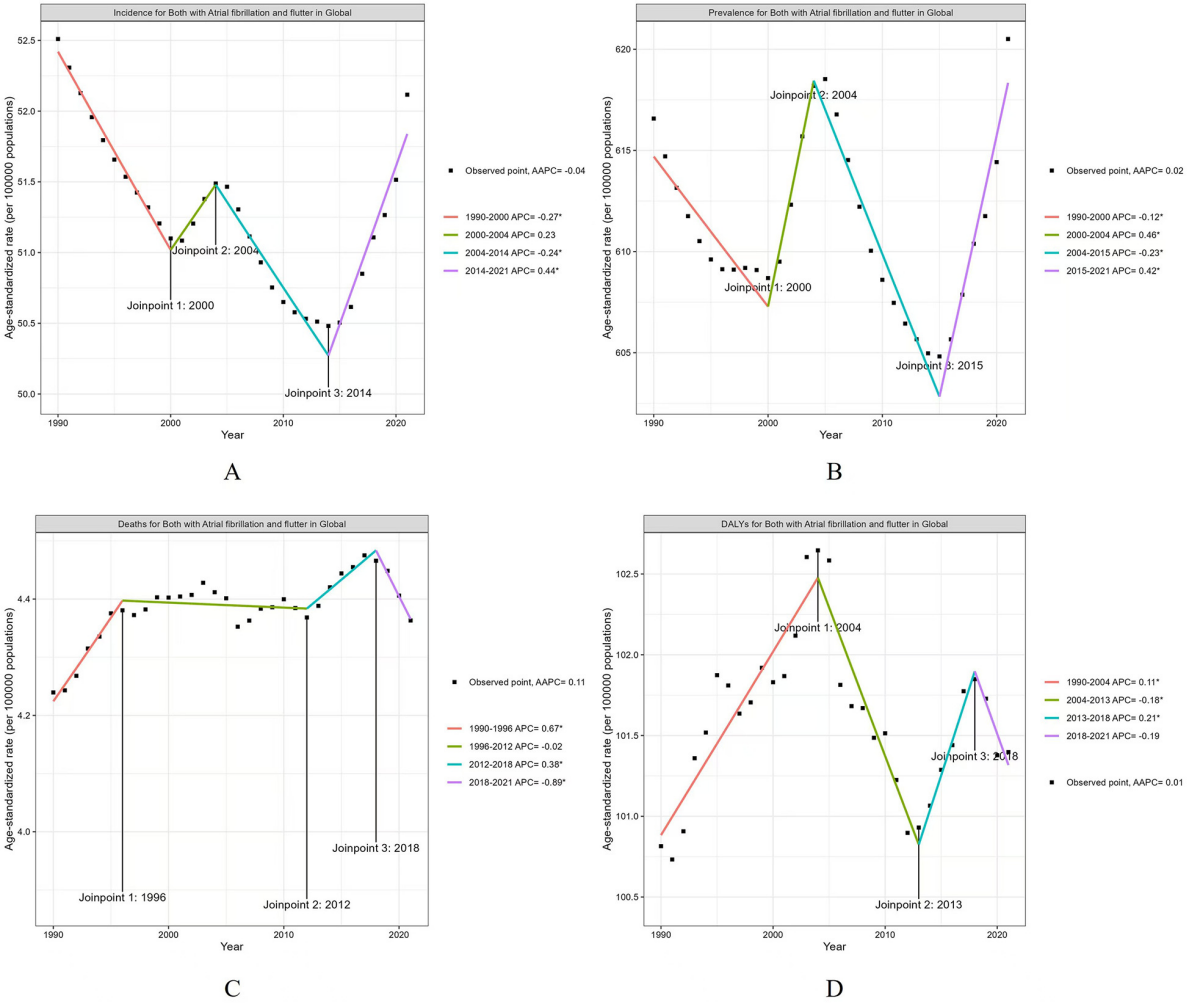
The APC of ASIR, ASPR, ASMR, and ASDR of AF/AFL in Global from 1990 to 2021 (* means *p*-values < 0.05 and significant results). **(A)** ASIR; **(B)** ASPR; **(C)** ASMR; **(D)** ASDR.

A comparison of the global burden of AF/AFL between 1990 and 2021 revealed notable fluctuations. The ASIR of AF/AFL declined significantly from 1990 to 2000 [Annual Percent Change (APC) = −0.27, *P* < 0.05] and from 2004 to 2014 (APC = −0.24, *P* < 0.05). However, it demonstrated a substantial upward trend from 2014 to 2021, with a notable increase (APC = 0.44, *P* < 0.05) (Table 1; Figure 4A).

**Table 1:** The AAPC of AF/AFL from 1990 to 2021.

Location	ASIR (95% CI)	ASPR (95% CI)	ASMR (95% CI)	ASDR (95% CI)
Global	−0.04 (−0.08 to 0.01)	0.02 (−0.02 to 0.06)	0.11 (0.02–0.2)	0.01 (−0.03 to 0.06)
SDI regions				
High SDI	0.04 (0–0.08)	0.12 (0.07–0.17)	−0.05 (−0.11 to 0.01)	−0.01 (−0.06 to 0.04)
High-middle SDI	−0.14 (−0.2 to −0.07)	−0.06 (−0.12 to 0.01)	0.1 (0.02–0.17)	−0.1 (−0.25 to 0.05)
Middle SDI	0.14 (0.1–0.19)	0.24 (0.21–0.27)	0 (−0.22 to 0.21)	0.11 (0.05–0.17)
Low-middle SDI	0.09 (0.09–0.1)	0.13 (0.12–0.14)	0.92 (0.61–1.22)	0.53 (0.35–0.71)
Low SDI	0.11 (0.09–0.13)	0.14 (0.12–0.17)	0.61 (0.14–1.08)	0.36 (0.12–0.6)
GBD super regions				
Central Europe, Eastern Europe, and Central Asia				
High-income	0.01 (−0.08 to 0.1)	0.02 (−0.07 to 0.1)	0.02 (−0.04 to 0.09)	0.01 (−0.05 to 0.07)
Latin America and Caribbean	−0.03 (−0.05 to −0.02)	0.02 (0.01–0.02)	−0.16 (−0.25 to −0.07)	−0.04 (−0.08 to 0.01)
North Africa and Middle East	0.1 (0.08–0.12)	0.19 (0.16–0.21)	0.45 (0.22–0.68)	0.22 (0.1–0.35)
South Asia	0.06 (0.03–0.1)	0.08 (0.05–0.1)	1.37 (0.59–2.15)	0.66 (0.41–0.91)
Southeast Asia, East Asia, and Oceania				
Sub-Saharan Africa	0.17 (0.15–0.2)	0.21 (0.19–0.24)	0.17 (0.1–0.23)	0.16 (0.13–0.19)
GBD regions				
Central Asia	0.04 (0.03–0.05)	0.15 (0.14–0.16)	0.73 (0.17–1.29)	0.38 (0.07–0.7)
Central Europe	0.26 (0.22–0.3)	0.27 (0.23–0.32)	−0.35 (−0.55 to −0.15)	−0.13 (−0.23 to −0.03)
Eastern Europe	0.26 (0.24–0.29)	0.32 (0.28–0.36)	0.49 (0.13–0.86)	0.43 (0.23–0.63)
Australasia	0.1 (0.01–0.19)	0.24 (0.14–0.34)	−0.24 (−0.45 to −0.03)	−0.14 (−0.22 to −0.06)
High-income Asia Pacific	−0.5 (−0.59 to −0.4)	−0.36 (−0.49 to −0.24)	−0.68 (−0.88 to −0.47)	−0.53 (−0.68 to −0.39)
High-income North America	0.42 (0.25–0.6)	0.43 (0.24–0.61)	0.86 (0.74–0.99)	0.57 (0.52–0.62)
Southern Latin America	−0.86 (−0.94 to −0.78)	−0.64 (−0.72 to −0.55)	0.05 (−0.24 to 0.35)	−0.35 (−0.53 to −0.17)
Western Europe	−0.05 (−0.15 to 0.06)	0.08 (0–0.16)	0.09 (−0.12 to 0.29)	0.05 (−0.05 to 0.15)
Andean Latin America	0.13 (0.11–0.15)	0.26 (0.24–0.28)	−0.43 (−0.68 to −0.19)	−0.13 (−0.26 to 0)
Caribbean	−0.05 (−0.06 to −0.03)	0.01 (−0.01 to 0.02)	−0.41 (−0.69 to −0.13)	−0.19 (−0.32 to −0.06)
Central Latin America	−0.02 (−0.03 to −0.01)	0.02 (0.01 to 0.03)	−0.29 (−0.57 to 0)	−0.05 (−0.15 to 0.05)
Tropical Latin America	−0.09 (−0.11 to −0.07)	−0.04 (−0.06 to −0.02)	−0.06 (−0.27 to 0.15)	0.01 (−0.09 to 0.11)
North Africa and Middle East				
South Asia				
East Asia	0.16 (0.04 to 0.28)	0.41 (0.3 to 0.51)	−0.36 (−0.62 to −0.1)	−0.14 (−0.29 to 0.02)
Oceania	0.04 (0.02 to 0.06)	0.12 (0.11 to 0.13)	−0.13 (−0.2 to −0.07)	−0.01 (−0.04 to 0.01)
Southeast Asia	0.05 (0.04 to 0.06)	0.13 (0.12 to 0.14)	0.69 (0.62 to 0.76)	0.38 (0.34 to 0.42)
Central Sub-Saharan Africa	−0.05 (−0.05 to −0.05)	−0.02 (−0.03 to −0.01)	0.44 (0.37 to 0.51)	0.24 (0.2 to 0.28)
Eastern Sub-Saharan Africa	0.23 (0.16 to 0.3)	0.3 (0.22 to 0.37)	0.12 (0.02 to 0.22)	0.14 (0.08 to 0.19)
Southern Sub-Saharan Africa	0.02 (0.01 to 0.03)	0.02 (0.01 to 0.02)	1.22 (1.05 to 1.39)	0.64 (0.39 to 0.88)
Western Sub-Saharan Africa	0.28 (0.26 to 0.29)	0.32 (0.31 to 0.33)	−0.04 (−0.12 to 0.04)	0.07 (0.03 to 0.11)
Countries/territories				
Afghanistan	0.13 (0.1 to 0.15)	0.27 (0.24 to 0.29)	0.55 (0.47 to 0.62)	0.41 (0.35 to 0.47)
Albania	−0.12 (−0.14 to −0.1)	0.04 (0.01 to 0.07)	0.71 (0.02 to 1.41)	0.24 (−0.19 to 0.66)
Algeria	0.12 (0.1 to 0.14)	0.24 (0.21 to 0.27)	0.56 (0.32 to 0.79)	0.3 (0.16 to 0.43)
American Samoa	0.18 (0.17 to 0.19)	0.26 (0.24 to 0.27)	0.27 (0.17 to 0.36)	0.28 (0.21 to 0.34)
Andorra	−0.44 (−0.47 to −0.4)	−0.38 (−0.42 to −0.35)	−0.95 (−1.44 to −0.47)	−0.69 (−0.88 to −0.5)
Angola	0.16 (0.15–0.17)	0.24 (0.23–0.25)	0.78 (0.68–0.89)	0.44 (0.38–0.5)
Antigua and Barbuda	0.01 (0–0.03)	0.09 (0.08–0.11)	0.05 (−0.48 to 0.59)	−0.06 (−0.39 to 0.27)
Argentina	−1.29 (−1.42 to −1.17)	−1.04 (−1.17 to −0.92)	−0.19 (−0.45 to 0.07)	−0.62 (−0.85 to −0.39)
Armenia	0.2 (0.18–0.21)	0.3 (0.29–0.32)	1.66 (0.29–3.05)	0.79 (0.12–1.46)
Australia	0.12 (0.04–0.21)	0.25 (0.16–0.34)	−0.25 (−0.55 to 0.06)	−0.16 (−0.25 to −0.07)
Austria	1.98 (1.87–2.09)	2.08 (1.98–2.18)	0.89 (0.44–1.35)	1.35 (1.17–1.53)
Azerbaijan	0.12 (0.11–0.13)	0.25 (0.23–0.28)	0.14 (−0.27–0.55)	0.23 (0.09–0.36)
Bahamas	−0.14 (−0.16 to −0.12)	−0.08 (−0.08 to −0.07)	0.33 (−0.52 to 1.18)	0.06 (−0.38 to 0.51)
Bahrain	0.08 (0.07–0.1)	0.22 (0.19–0.25)	−0.71 (−1.33 to −0.09)	−0.63 (−1.06 to −0.19)
Bangladesh	0.06 (0.03–0.08)	0.14 (0.08–0.2)	1.68 (0.85–2.53)	0.82 (0.37–1.26)
Barbados	0.08 (0.06–0.1)	0.16 (0.14–0.18)	0.12 (−0.2 to 0.44)	0 (−0.22 to 0.23)
Belarus	0.26 (0.23–0.28)	0.35 (0.33–0.36)	0.43 (0.16–0.69)	0.38 (0.12–0.64)
Belgium	−0.53 (−0.65 to −0.41)	−0.18 (−0.28 to −0.07)	−0.17 (−0.77 to 0.43)	−0.11 (−0.46 to 0.25)
Belize	0.04 (0.02–0.07)	0.12 (0.09–0.14)	0.49 (−0.32 to 1.3)	0.22 (−0.13 to 0.56)
Benin	0.12 (0.11–0.13)	0.16 (0.15–0.17)	0.53 (0.44–0.62)	0.32 (0.28–0.37)
Bermuda	−0.1 (−0.12 to −0.08)	−0.02 (−0.04 to −0.01)	−1.34 (−1.52 to −1.16)	−0.84 (−0.93 to −0.76)
Bhutan	0.05 (0.03–0.07)	0.18 (0.16–0.2)	1.73 (1.66–1.8)	0.87 (0.82–0.92)
Bolivia (Plurinational State of)	0.02 (0.01–0.03)	0.14 (0.13–0.15)	0.1 (0.02–0.18)	0.05 (0–0.09)
Bosnia and Herzegovina	0.02 (0–0.04)	0.15 (0.1–0.19)	0.9 (0.46–1.35)	0.37 (0.15–0.59)
Botswana	0.17 (0.15–0.18)	0.22 (0.2–0.23)	−0.01 (−0.68 to 0.67)	0 (−0.53 to 0.53)
Brazil	−0.09 (−0.1 to −0.07)	−0.04 (−0.06 to −0.02)	−0.07 (−0.29 to 0.14)	0.04 (−0.04 to 0.11)
Brunei Darussalam	−0.62 (−0.64 to −0.6)	−0.55 (−0.57 to −0.53)	−0.41 (−0.77 to −0.06)	−0.56 (−0.79 to −0.33)
Bulgaria	−0.11 (−0.15 to −0.07)	−0.07 (−0.12 to −0.03)	0.42 (−0.9 to 1.75)	0.39 (0.22–0.57)
Burkina Faso	0.14 (0.11–0.16)	0.16 (0.15–0.17)	1.02 (0.88–1.17)	0.7 (0.61–0.79)
Burundi	0.04 (0.03–0.04)	0.09 (0.08–0.09)	0 (−0.1 to 0.1)	−0.08 (−0.13 to −0.04)
Cabo Verde	0.13 (0.12–0.14)	0.18 (0.17–0.19)	1.22 (0.7–1.75)	0.73 (0.41–1.05)
Cambodia	−0.02 (−0.03 to −0.01)	0.09 (0.08–0.1)	0.99 (0.95–1.03)	0.43 (0.4–0.47)
Cameroon	0.34 (0.33–0.35)	0.4 (0.38–0.42)	0.16 (0.05–0.26)	0.24 (0.1–0.38)
Canada	−0.65 (−0.78 to −0.53)	−0.55 (−0.68 to −0.41)	−0.28 (−0.47 to −0.08)	−0.61 (−0.71 to −0.52)
Central African Republic	−0.02 (−0.02 to −0.01)	0 (−0.01 to 0)	0.12 (−0.04 to 0.28)	0.04 (−0.02 to 0.11)
Chad	0.11 (0.1–0.13)	0.15 (0.14–0.17)	0.84 (0.81–0.88)	0.56 (0.51–0.62)
Chile	−0.35 (−0.38 to −0.31)	−0.2 (−0.23 to −0.16)	0.27 (−0.21 to 0.76)	0.05 (−0.15 to 0.25)
China	0.17 (0.05–0.29)	0.43 (0.32–0.53)	−0.34 (−0.62 to −0.05)	−0.13 (−0.3 to 0.05)
Colombia	−0.07 (−0.09 to −0.06)	0.01 (−0.01 to 0.03)	−0.51 (−1.05 to 0.04)	−0.22 (−0.43 to 0)
Comoros	0.09 (0.07–0.1)	0.14 (0.13–0.15)	−0.1 (−0.19 to 0)	−0.13 (−0.18 to −0.07)
Congo	0.13 (0.12–0.13)	0.2 (0.19–0.21)	0.08 (−0.02 to 0.19)	0 (−0.11 to 0.11)
Cook Islands	0.33 (0.32–0.35)	0.45 (0.43–0.46)	−0.42 (−0.5 to −0.35)	−0.23 (−0.28 to −0.18)
Costa Rica	−0.1 (−0.11 to −0.08)	−0.06 (−0.07 to −0.05)	−0.19 (−0.77 to 0.4)	−0.12 (−0.33 to 0.09)
Croatia	0.52 (0.27–0.78)	0.39 (0.15–0.64)	0.56 (0.16–0.95)	0.35 (0.09–0.61)
Cuba	−0.09 (−0.11 to −0.06)	−0.05 (−0.07 to −0.03)	−0.29 (−0.55 to −0.02)	−0.17 (−0.29 to −0.04)
Cyprus	−0.9 (−0.99 to −0.8)	−0.95 (−1.04 to −0.86)	−1.81 (−2.41 to −1.2)	−1.76 (−2.17 to −1.35)
Czechia	1.3 (1.12–1.48)	1.26 (1.07–1.45)	0.65 (0.44–0.85)	0.87 (0.65–1.09)
Côte d'Ivoire	0.11 (0.1 –0.13)	0.17 (0.16–0.18)	0.34 (0.23–0.46)	0.27 (0.19–0.35)
Democratic People's Republic of Korea	−0.11 (−0.12 to −0.09)	−0.01 (−0.03 to 0.01)	0 (−0.1 to 0.1)	−0.01 (−0.06 to 0.05)
Democratic Republic of the Congo	−0.15 (−0.15 to −0.14)	−0.14 (−0.15 to −0.13)	0.45 (0.36–0.53)	0.24 (0.19–0.28)
Denmark	−0.03 (−0.12 to 0.06)	0.23 (0.04–0.43)	0.79 (0.56–1.02)	0.4 (0.22–0.59)
Djibouti	0.23 (0.21–0.24)	0.29 (0.27–0.3)	0.06 (0.01–0.1)	0.06 (0.02–0.1)
Dominica	0.05 (0.03–0.07)	0.1 (0.08–0.11)	0.02 (−0.01 to 0.06)	0.06 (0.04–0.09)
Dominican Republic	−0.04 (−0.05 to −0.03)	0.02 (0.01–0.03)	−0.45 (−0.97 to 0.08)	−0.13 (−0.38 to 0.12)
East Asia	0.16 (0.04–0.28)	0.41 (0.3–0.51)	−0.36 (−0.62 to −0.1)	−0.14 (−0.29 to 0.02)
Ecuador	−0.04 (−0.06 to −0.02)	0.05 (0.03–0.07)	−0.78 (−1.2 to −0.36)	−0.41 (−0.71 to −0.11)
Egypt	0.36 (0.33–0.38)	0.52 (0.5–0.55)	−0.36 (−0.63 to −0.09)	0 (−0.15 to 0.16)
El Salvador	−0.04 (−0.05 to −0.03)	0.06 (0.05–0.08)	0.2 (−0.39 to 0.81)	0.17 (−0.19 to 0.53)
Equatorial Guinea	0.36 (0.34–0.38)	0.53 (0.51–0.55)	0.85 (0.77–0.93)	0.52 (0.46–0.59)
Eritrea	0.1 (0.08–0.12)	0.16 (0.12–0.19)	0.74 (0.69–0.79)	0.29 (0.24–0.34)
Estonia	0.31 (0.3–0.32)	0.42 (0.41–0.42)	0.94 (0.74–1.15)	0.62 (0.41–0.83)
Eswatini	0.13 (0.11–0.14)	0.16 (0.15–0.18)	0.2 (−0.02 to 0.43)	0.22 (0.12–0.31)
Ethiopia	0.41 (0.36–0.46)	0.54 (0.49–0.58)	−0.03 (−0.11 to 0.04)	0.05 (0–0.09)
Fiji	0.3 (0.29–0.32)	0.35 (0.34–0.36)	0.79 (0.43–1.15)	0.48 (0.31–0.64)
Finland	−1.08 (−1.23 to −0.93)	−1.09 (−1.27 to −0.91)	−1.59 (−2.07 to −1.11)	−1.37 (−1.54 to −1.19)
France	−0.5 (−0.52 to −0.47)	−0.37 (−0.4 to −0.34)	−0.61 (−0.73 to −0.49)	−0.56 (−0.63 to −0.49)
Gabon	0.16 (0.14–0.17)	0.23 (0.22–0.24)	0.34 (0.25–0.43)	0.22 (0.16–0.28)
Gambia	0.08 (0.06–0.11)	0.13 (0.12–0.15)	0.92 (0.8–1.04)	0.58 (0.48–0.68)
Georgia	−0.01 (−0.03 to 0.02)	0.06 (0.04–0.07)	1.98 (0.45–3.54)	1.17 (0.38–1.97)
Germany	0.09 (0.04–0.14)	0.51 (0.45–0.57)	0.66 (0.35–0.97)	0.49 (0.28–0.7)
Ghana	0.27 (0.23–0.32)	0.33 (0.28–0.38)	−0.05 (−0.13 to 0.03)	0.01 (−0.05 to 0.07)
Greece	−0.26 (−0.61 to 0.08)	−0.27 (−0.64 to 0.11)	−0.21 (−0.4 to −0.02)	−0.23 (−0.57 to 0.11)
Greenland	−0.3 (−0.39 to −0.21)	−0.17 (−0.27 to −0.07)	−0.68 (−0.87 to −0.5)	−0.63 (−0.73 to −0.53)
Grenada	0.01 (−0.01 to 0.03)	0.05 (0.03–0.07)	0.79 (0.36–1.22)	0.44 (0.18–0.7)
Guam	0.33 (0.31–0.35)	0.39 (0.37 –0.4)	−4.23 (−5.15 to −3.31)	−1.53 (−1.85 to −1.21)
Guatemala	0.06 (0.05–0.08)	0.18 (0.16–0.19)	−0.99 (−1.9 to −0.07)	−0.4 (−0.99 to 0.2)
Guinea	0.1 (0.09–0.11)	0.14 (0.13–0.15)	0.67 (0.61–0.73)	0.45 (0.41–0.5)
Guinea-Bissau	0.08 (0.07–0.09)	0.13 (0.11–0.14)	0.52 (0.41–0.63)	0.31 (0.25–0.37)
Guyana	−0.07 (−0.1 to −0.05)	−0.02 (−0.04 to 0)	0.96 (0.12–1.81)	0.46 (0.09–0.82)
Haiti	−0.02 (−0.04 to 0)	0.08 (0.07–0.1)	−0.05 (−0.15 to 0.04)	−0.04 (−0.09 to 0.01)
Honduras	−0.06 (−0.08 to −0.05)	0.02 (0–0.04)	1.85 (1.05–2.65)	1.17 (0.84–1.5)
Hungary	−0.62 (−0.63 to −0.6)	−0.55 (−0.56 to −0.53)	−0.6 (−0.86 to −0.33)	−0.46 (−0.53 to −0.39)
Iceland	0.26 (0.23–0.3)	0.39 (0.37–0.41)	0.58 (0.31–0.85)	0.33 (0.22–0.44)
India	0.08 (0.04–0.11)	0.08 (0.04–0.12)	1.32 (0.28–2.37)	0.65 (0.21–1.1)
Indonesia	0.1 (0.09–0.1)	0.12 (0.12–0.13)	1.87 (1.8–1.94)	0.87 (0.82–0.92)
Iran (Islamic Republic of)	0.26 (0.22–0.3)	0.32 (0.26–0.38)	−0.03 (−0.17 to 0.11)	0.06 (−0.03 to 0.15)
Iraq	0.15 (0.14–0.16)	0.27 (0.26–0.29)	1.32 (1.15–1.49)	0.64 (0.54–0.75)
Ireland	−0.65 (−0.97 to −0.33)	−0.51 (−0.73 to −0.3)	−0.31 (−0.94 to 0.33)	−0.63 (−0.97 to −0.28)
Israel	0.81 (0.43–1.18)	0.96 (0.61–1.32)	−0.88 (−1.23 to −0.52)	0.14 (−0.17 to 0.44)
Italy	−0.37 (−0.53 to −0.21)	−0.35 (−0.49 to −0.22)	0.38 (0.05–0.71)	−0.16 (−0.36 to 0.04)
Jamaica	0.08 (0.07–0.1)	0.14 (0.13–0.15)	0.21 (−0.23 to 0.66)	0.2 (−0.02 to 0.41)
Japan	−0.84 (−0.97 to −0.7)	−0.85 (−0.99 to −0.72)	−0.86 (−1.1 to −0.62)	−0.85 (−1.04 to −0.66)
Jordan	0.24 (0.23–0.25)	0.37 (0.35–0.39)	−0.51 (−1.64 to 0.63)	−0.28 (−0.68 to 0.12)
Kazakhstan	0.04 (0.02–0.07)	0.15 (0.13–0.17)	0.9 (0.45–1.34)	0.41 (0.23–0.59)
Kenya	0.09 (0.08–0.1)	0.07 (0.04–0.09)	1.1 (0.97–1.22)	0.58 (0.5–0.67)
Kiribati	0.04 (0.02–0.06)	0.12 (0.1–0.13)	0.62 (0.59–0.66)	0.28 (0.26–0.31)
Kuwait	0.2 (0.18–0.23)	0.31 (0.29–0.33)	0.86 (−0.64 to 2.39)	0.4 (−0.36 to 1.17)
Kyrgyzstan	−0.04 (−0.07 to −0.01)	0.07 (0.04–0.09)	0.05 (−0.5 to 0.59)	0.11 (−0.14 to 0.35)
Lao People's Democratic Republic	−0.04 (−0.05 to −0.03)	0.1 (0.09–0.11)	0.66 (0.59–0.72)	0.27 (0.22–0.32)
Latvia	0.69 (0.4–0.98)	0.81 (0.52–1.11)	0.65 (0.2–1.09)	0.68 (0.48–0.88)
Lebanon	0.27 (0.25–0.29)	0.4 (0.38–0.41)	−1.24 (−1.51 to −0.96)	−0.99 (−1.13 to −0.84)
Lesotho	0.14 (0.12–0.16)	0.16 (0.15–0.17)	1.36 (0.88–1.83)	0.93 (0.68–1.18)
Liberia	0.03 (0.02–0.04)	0.09 (0.08–0.1)	0.26 (0.15–0.38)	0.19 (0.06–0.32)
Libya	0.16 (0.15–0.17)	0.25 (0.24–0.27)	0.67 (0.47–0.87)	0.49 (0.35–0.62)
Lithuania	0.31 (0.29–0.32)	0.37 (0.35–0.38)	0.69 (0.38–1)	0.57 (0.42–0.71)
Luxembourg	−0.09 (−0.12 to −0.06)	−0.03 (−0.06 to 0.01)	−0.16 (−0.78 to 0.47)	−0.25 (−0.67 to 0.18)
Madagascar	0.16 (0.14–0.18)	0.2 (0.19–0.21)	0.12 (0.03–0.22)	0.08 (0.01––0.15)
Malawi	0.24 (0.22–0.25)	0.29 (0.27–0.31)	0.7 (0.5–0.9)	0.54 (0.45–0.62)
Malaysia	0.09 (0.07–0.11)	0.2 (0.18–0.23)	1.31 (1.01–1.6)	0.66 (0.52–0.8)
Maldives	0.03 (0.02–0.04)	0.09 (0.08–0.1)	−0.08 (−0.23 to 0.07)	−0.15 (−0.22 to −0.08)
Mali	0.12 (0.11–0.14)	0.19 (0.18–0.2)	0.13 (0.03–0.23)	0.16 (0.12–0.21)
Malta	−0.38 (−0.53 to −0.22)	−0.38 (−0.5 to −0.26)	−0.4 (−0.69 to −0.11)	−0.47 (−0.72 to −0.21)
Marshall Islands	0.07 (0.06–0.08)	0.2 (0.19–0.21)	0.2 (0.14–0.26)	0.23 (0.18–0.29)
Mauritania	0.17 (0.16–0.18)	0.29 (0.28–0.3)	0.34 (0.24–0.43)	0.21 (0.17–0.26)
Mauritius	0.01 (0–0.02)	0.1 (0.09–0.11)	−0.31 (−0.98 to 0.37)	−0.23 (−0.46 to 0.01)
Mexico	0.06 (0.04–0.08)	0.06 (0.05–0.08)	−0.47 (−0.92 to −0.01)	−0.12 (−0.22 to −0.02)
Micronesia (Federated States of)	−0.01 (−0.02 to 0)	0.07 (0.06–0.08)	0.19 (0.16–0.22)	0.12 (0.1–0.14)
Monaco	−0.41 (−0.44 to −0.39)	−0.3 (−0.32 to −0.27)	0.01 (−0.06 to 0.07)	−0.24 (−0.29 to −0.18)
Mongolia	0.1 (0.08–0.13)	0.22 (0.21–0.23)	−0.35 (−0.62 to −0.08)	−0.09 (−0.16 to −0.01)
Montenegro	−0.18 (−0.21 to −0.15)	−0.12 (−0.15 to −0.08)	1.68 (0.87–2.49)	1.03 (0.55–1.52)
Morocco	0.08 (0.06–0.1)	0.2 (0.18–0.22)	1.09 (1.01–1.16)	0.68 (0.63–0.74)
Mozambique	0.19 (0.18–0.2)	0.23 (0.23–0.24)	0.97 (0.8–1.14)	0.73 (0.66–0.8)
Myanmar	−0.1 (−0.11 to −0.09)	0 (−0.01 to 0.02)	0.69 (0.61–0.77)	0.25 (0.21–0.28)
Namibia	0.03 (0.02–0.03)	0.09 (0.08–0.09)	0.71 (0.55–0.86)	0.41 (0.31–0.51)
Nauru	0.16 (0.14–0.17)	0.14 (0.12–0.16)	0.76 (0.72–0.8)	0.41 (0.38–0.44)
Nepal	−0.08 (−0.1 to −0.06)	0.01 (−0.01 to 0.03)	1.83 (1.75–1.91)	0.86 (0.8–0.92)
Netherlands	−0.42 (−0.51 to −0.33)	−0.27 (−0.35 to −0.2)	−0.51 (−0.67 to −0.35)	−0.41 (−0.5 to −0.31)
New Zealand	−0.04 (−0.08 to 0)	0.17 (0.09–0.25)	−0.26 (−0.52 to 0)	−0.16 (−0.34 to 0.02)
Nicaragua	−0.06 (−0.08 to −0.05)	0.02 (0.01–0.02)	0.24 (−0.24 to 0.72)	0.07 (−0.18 to 0.33)
Niger	0.07 (0.05–0.09)	0.13 (0.12–0.15)	0.36 (0.29–0.43)	0.2 (0.16–0.25)
Nigeria	0.43 (0.42–0.44)	0.47 (0.45–0.49)	−0.41 (−0.5 to −0.33)	−0.13 (−0.17 to −0.08)
Niue	0.2 (0.18–0.22)	0.31 (0.29–0.33)	0.04 (−0.03 to 0.11)	0.08 (0.01–0.16)
North Macedonia	−0.18 (−0.22 to −0.14)	−0.1 (−0.14 to −0.06)	1.88 (1.4–2.37)	0.87 (0.49–1.25)
Northern Mariana Islands	0.1 (0.07–0.13)	0.14 (0.12–0.17)	0.41 (0.13–0.69)	0.32 (0.2–0.44)
Norway	−0.33 (−0.4 to −0.27)	−0.16 (−0.23 to −0.09)	−0.29 (−1.21 to 0.64)	−0.41 (−0.97 to 0.15)
Oman	0.55 (0.52–0.59)	0.71 (0.7–0.73)	0.48 (−0.16 to 1.12)	0.4 (0.02–0.78)
Pakistan	0.04 (0.03–0.05)	0.03 (0.02–0.04)	1.62 (1.49–1.74)	0.8 (0.73–0.87)
Palau	0.21 (0.2–0.22)	0.32 (0.31–0.33)	−0.14 (−0.24 to −0.04)	−0.05 (−0.09 to −0.01)
Palestine	0.12 (0.11 –0.13)	0.23 (0.21–0.24)	0.07 (−0.14 to 0.28)	0.02 (−0.14 to 0.18)
Panama	0.01 (−0.01 to 0.02)	0.07 (0.05–0.09)	0.31 (−0.04 to 0.65)	0.17 (0–0.34)
Papua New Guinea	0.01 (−0.02 to 0.03)	0.07 (0.05–0.09)	0.39 (0.3–0.48)	0.18 (0.15–0.21)
Paraguay	−0.16 (−0.18 to −0.15)	−0.11 (−0.13 to −0.1)	0.59 (0.18–1.01)	0.28 (0.09–0.46)
Peru	0.23 (0.21–0.26)	0.38 (0.35–0.4)	−0.48 (−0.94 to −0.01)	−0.06 (−0.29 to 0.17)
Philippines	0.04 (0.03–0.05)	0.03 (0.02–0.03)	−0.32 (−0.71 to 0.07)	−0.05 (−0.2 to 0.1)
Poland	0.81 (0.64–0.98)	0.93 (0.79–1.06)	−1.33 (−1.77 to −0.89)	−0.3 (−0.72 to 0.12)
Portugal	−0.33 (−0.4 to −0.25)	−0.28 (−0.32 to −0.25)	−0.93 (−1.27 to −0.59)	−0.57 (−0.77 to −0.37)
Puerto Rico	−0.03 (−0.05 to −0.02)	0.03 (0.01–0.04)	−1.47 (−1.87 to −1.06)	−0.66 (−0.88 to −0.44)
Qatar	0.22 (0.19–0.25)	0.33 (0.3–0.36)	−2.11 (−2.92 to −1.3)	−1.43 (−1.98 to −0.89)
Republic of Korea	−0.05 (−0.18 to 0.08)	0.39 (0.27–0.52)	0.12 (−0.09 to 0.32)	0.1 (−0.01 to 0.21)
Republic of Moldova	0.32 (0.3–0.33)	0.4 (0.38–0.41)	−0.54 (−0.83 to −0.25)	0.11 (−0.05 to 0.27)
Romania	−1.08 (−1.24 to −0.92)	−1.03 (−1.2 to −0.86)	−0.2 (−0.47 to 0.08)	−0.51 (−0.72 to −0.29)
Russian Federation	0.29 (0.25–0.33)	0.35 (0.29–0.4)	0.49 (0.02–0.96)	0.48 (0.23–0.74)
Rwanda	0.03 (0.02–0.04)	0.12 (0.1–0.14)	−0.73 (−0.92 to −0.53)	−0.56 (−0.7 to −0.41)
Saint Kitts and Nevis	−0.08 (−0.11 to −0.04)	0.02 (−0.01 to 0.06)	−0.16 (−0.83 to 0.51)	−0.22 (−0.59 to 0.15)
Saint Lucia	−0.11 (−0.15 to −0.08)	−0.03 (−0.06 to −0.01)	−1.31 (−1.76 to −0.86)	−0.85 (−1.05 to −0.64)
Saint Vincent and the Grenadines	0.1 (0.09–0.11)	0.19 (0.18–0.2)	−0.13 (−0.75 to 0.5)	−0.23 (−0.69 to 0.23)
Samoa	0.03 (0.02–0.05)	0.13 (0.11–0.14)	−0.02 (−0.07 to 0.03)	0.05 (0.02–0.09)
San Marino	−0.46 (−0.48 to −0.43)	−0.35 (−0.37 to −0.32)	−2.44 (−2.91 to −1.97)	−1.33 (−1.55 to −1.11)
Sao Tome and Principe	0.22 (0.21–0.23)	0.32 (0.31–0.33)	1.12 (0.98–1.26)	0.8 (0.74–0.87)
Saudi Arabia	0.4 (0.38–0.42)	0.54 (0.51–0.57)	0.4 (0.24–0.56)	0.41 (0.32–0.5)
Senegal	0.05 (0.03–0.06)	0.1 (0.09–0.11)	0.85 (0.73–0.96)	0.5 (0.41–0.59)
Serbia	−0.92 (−1.03 to −0.81)	−0.97 (−1.08 to −0.85)	−0.76 (−1.16 to −0.36)	−0.73 (−0.93 to −0.53)
Seychelles	−0.02 (−0.03 to −0.01)	0.08 (0.06–0.1)	0.19 (−0.16 to 0.54)	−0.03 (−0.18 to 0.12)
Sierra Leone	−0.06 (−0.07 to −0.05)	−0.02 (−0.04 to −0.01)	0.35 (0.31–0.4)	0.18 (0.16–0.2)
Singapore	−0.58 (−0.62 to −0.53)	−0.45 (−0.5 to −0.4)	−1.51 (−1.76 to −1.26)	−0.97 (−1.1 to −0.85)
Slovakia	0.34 (0.3–0.38)	0.23 (0.2–0.27)	0.21 (−0.09 to 0.52)	0.13 (−0.01 to 0.27)
Slovenia	−0.07 (−0.13 to −0.02)	0.05 (0–0.11)	0.2 (−0.44 to 0.85)	−0.06 (−0.43 to 0.31)
Solomon Islands	0.05 (0.04–0.06)	0.1 (0.09–0.11)	0.62 (0.56–0.67)	0.36 (0.26–0.45)
Somalia	0.13 (0.12–0.14)	0.15 (0.14–0.16)	−0.67 (−0.8 to −0.54)	−0.3 (−0.35 to −0.24)
South Africa	−0.03 (−0.03 to −0.02)	−0.04 (−0.04 to −0.03)	1.34 (1.05–1.64)	0.62 (0.39–0.86)
South Sudan	0.21 (0.18–0.23)	0.27 (0.24–0.29)	−0.32 (−0.41 to −0.24)	−0.15 (−0.21 to −0.1)
Spain	−0.22 (−0.51 to 0.07)	−0.03 (−0.44 to 0.4)	−0.41 (−0.74 to −0.08)	−0.31 (−0.49 to −0.12)
Sri Lanka	0.09 (0.08–0.1)	0.16 (0.15–0.17)	0.42 (−0.08 to 0.93)	0.21 (−0.04 to 0.47)
Sudan	0.37 (0.36–0.38)	0.52 (0.51–0.52)	0.36 (0.31–0.42)	0.36 (0.32–0.4)
Suriname	−0.09 (−0.11 to −0.08)	−0.05 (−0.06 to −0.04)	0.14 (−0.01 to 0.29)	−0.01 (−0.61 to 0.6)
Sweden	1.14 (0.7–1.59)	1.29 (0.93–1.66)	2.37 (1.31–3.45)	1.45 (0.83–2.07)
Switzerland	−0.22 (−0.5 to 0.05)	−0.03 (−0.36 to 0.3)	0.39 (−0.02 to 0.81)	−0.01 (−0.22 to 0.2)
Syrian Arab Republic	0.11 (0.1–0.11)	0.26 (0.25–0.27)	0.06 (−0.69 to 0.82)	0.11 (−0.18 to 0.41)
Taiwan (Province of China)	−0.09 (−0.13 to −0.05)	−0.04 (−0.12 to 0.03)	−0.91 (−1.71 to −0.11)	−0.63 (−0.84 to −0.42)
Tajikistan	−0.01 (−0.03–0.01)	0.09 (0.07–0.11)	−0.78 (−1.66 to 0.1)	−0.16 (−0.42 to 0.1)
Thailand	0.04 (0.02–0.06)	0.15 (0.13–0.16)	−0.78 (−1.02 to −0.55)	−0.35 (−0.48 to −0.22)
Timor-Leste	0.01 (0.01–0.02)	0.11 (0.1–0.12)	0.81 (0.73–0.88)	0.38 (0.34–0.43)
Togo	0.04 (0.03–0.05)	0.06 (0.05–0.07)	0.62 (0.49–0.74)	0.39 (0.3–0.48)
Tokelau	0.27 (0.25–0.28)	0.39 (0.37–0.4)	−0.07 (−0.2 to 0.05)	−0.08 (−0.11 to −0.04)
Tonga	0.21 (0.2–0.22)	0.28 (0.27–0.29)	0.42 (0.21–0.63)	0.28 (0.17–0.4)
Trinidad and Tobago	0.02 (0.01–0.03)	0.1 (0.09–0.11)	−1.01 (−1.27 to −0.75)	−0.37 (−0.72 to −0.02)
Tunisia	0.15 (0.14–0.16)	0.26 (0.25–0.28)	0.74 (0.51–0.98)	0.45 (0.3–0.59)
Turkey	−0.53 (−0.57 to −0.48)	−0.55 (−0.6 to −0.51)	0.44 (−0.39 to 1.27)	−0.08 (−0.39 to 0.22)
Turkmenistan	0.27 (0.26–0.29)	0.38 (0.37–0.39)	0.88 (0.44–1.31)	0.65 (0.5–0.81)
Tuvalu	0.19 (0.18–0.21)	0.34 (0.32–0.36)	0.07 (0.06–0.09)	0.04 (0.02–0.06)
Uganda	0.03 (0.01–0.06)	0.07 (0.05–0.09)	0.28 (0.18–0.37)	0.11 (0.07–0.16)
Ukraine	0.11 (0.09–0.14)	0.16 (0.13–0.18)	0.26 (−0.43 to 0.96)	0.11 (−0.18 to 0.39)
United Arab Emirates	0.22 (0.2–0.24)	0.35 (0.33–0.37)	0.08 (−2.03 to 2.25)	0.13 (−1.27 to 1.55)
United Kingdom	−0.06 (−0.11 to −0.01)	0.14 (0.09–0.18)	0.22 (−0.06 to 0.5)	0.12 (0–0.23)
United Republic of Tanzania	0.44 (0.33–0.55)	0.5 (0.4–0.61)	0.01 (−0.09 to 0.1)	0.12 (0.01–0.23)
United States Virgin Islands	0.01 (0.01–0.02)	0.07 (0.06–0.08)	−1.33 (−1.77 to −0.88)	−0.9 (−1.11 to −0.68)
United States of America	0.56 (0.44–0.68)	0.58 (0.4–0.75)	1 (0.86–1.15)	0.69 (0.63–0.74)
Uruguay	−0.33 (−0.35 to −0.3)	−0.21 (−0.23 to −0.19)	0.43 (0.25–0.62)	0.15 (0.04–0.27)
Uzbekistan	0.15 (0.12–0.18)	0.25 (0.21–0.29)	0.7 (0.01–1.4)	0.46 (0.29–0.62)
Vanuatu	0.04 (0.03–0.06)	0.08 (0.06–0.09)	0.33 (0.3–0.36)	0.17 (0.07–0.26)
Venezuela (Bolivarian Republic of)	−0.23 (−0.24 to −0.21)	−0.18 (−0.2 to −0.17)	0.2 (−0.12 to 0.52)	0 (−0.13 to 0.13)
Viet Nam	0.19 (0.16–0.23)	0.33 (0.3–0.36)	1.16 (1.09–1.22)	0.71 (0.68–0.74)
Yemen	0.19 (0.17–0.2)	0.3 (0.29–0.31)	0.6 (0.54–0.67)	0.43 (0.38–0.49)
Zambia	0.07 (0.06–0.08)	0.14 (0.12–0.15)	1.12 (0.92–1.31)	0.88 (0.76–1)

ASIR, age-standardized incidence rate; ASPR, age-standardized prevalence rate; ASMR, age-standardized mortality rate; ASDR, age-standardized DALYs rate; AAPC, average annual percent change; CI, confidence interval.

The ASPR for AF/AFL exhibited a global decline from 1990 to 2000 (APC = −0.12, *P* < 0.05). This was followed by an increase from 2000 to 2004 (APC = 0.46, *P* < 0.05), a decline from 2004 to 2015 (APC = −0.23, *P* < 0.05), and another increase from 2015 to 2021 (APC = 0.42, *P* < 0.05) (Table 1; Figure 4B).

The ASMR for AF/AFL showed an upward trend from 1990 to 1996 (APC = 0.67, *P* < 0.05), followed by a further increase from 2012 to 2018 (APC = 0.38, *P* < 0.05). However, it decreased from 2018 to 2021 (APC = −0.89, *P* < 0.05) (Table 1; Figure 4C).

The ASDR for AF/AFL showed a significant increase from 1990 to 2004 (APC = 0.11, *P* < 0.05), followed by a decrease from 2004 to 2013 (APC = −0.18, *P* < 0.05). There was an increase in ASDR from 2013 to 2018 (APC = 0.21, *P* < 0.05), followed by a decrease from 2018 to 2021 (APC = −0.19, *P* < 0.05) (Table 1; Figure 4D).

### Trends in specific SDI regions

**Figure 5:**
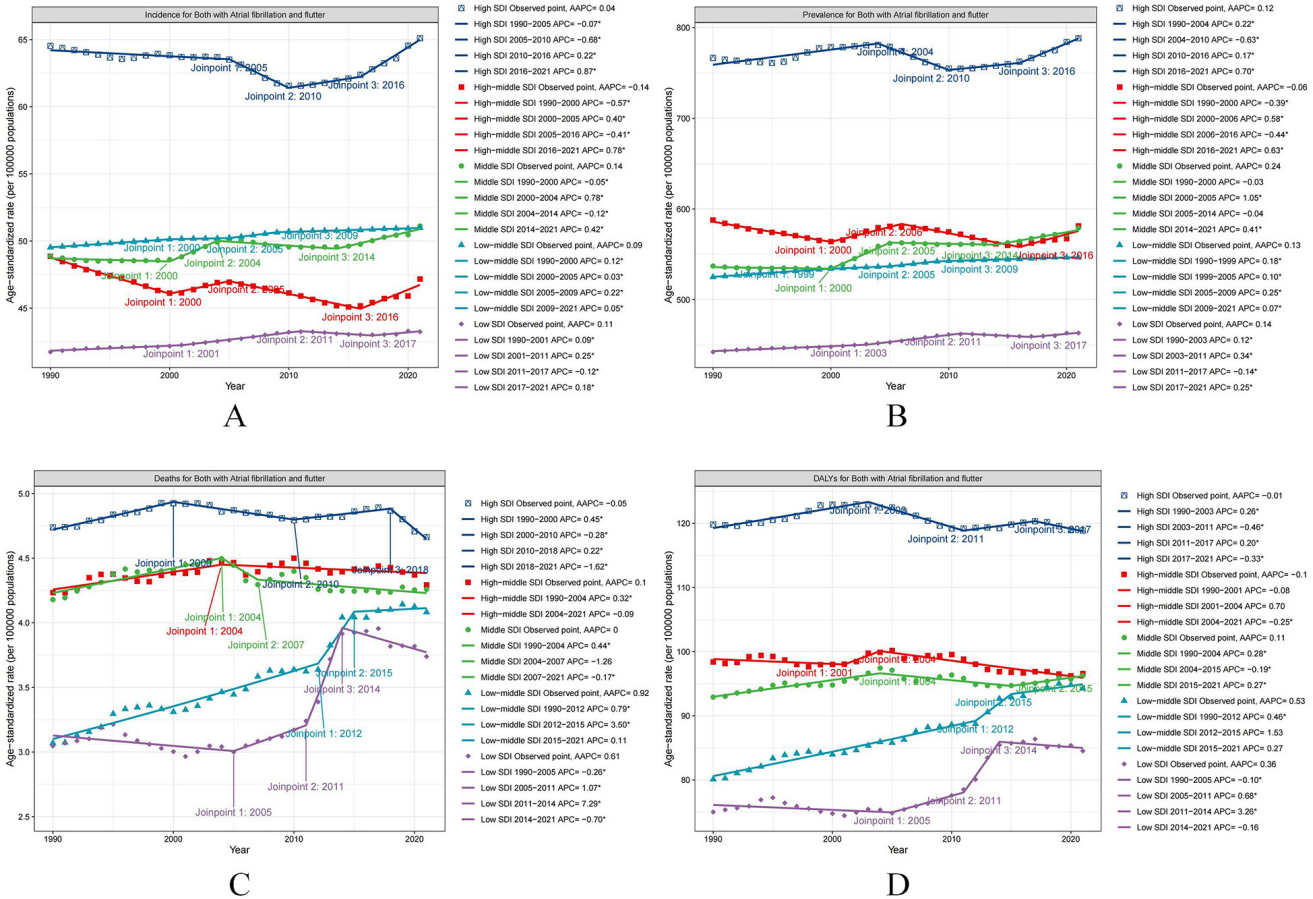
The APC of ASIR, ASPR, ASMR, and ASDR of AF/AFL in five SDI regions from 1990 to 2021 (* means *p*-values < 0.05 and significant results). **(A)** ASIR; **(B)** ASPR; **(C)** ASMR; **(D)** ASDR.

Across different SDI regions, the ASIR of AF/AFL showed increasing trends in high SDI, middle SDI, low-middle SDI, and low SDI regions, with the average annual percent changes (AAPCs) being 0.04 (95% CI: 0.0, 0.08), 0.14 (95% CI: 0.1, 0.19), 0.09 (95% CI: 0.09, 0.1), and 0.11 (95% CI: 0.09, 0.13) from 1990 to 2021, respectively (Table 1; Figure 5A). However, high-middle SDI regions showed a downward trend with an AAPC of −0.14 (95% CI: −0.2, −0.07) (Table 1; Figure 5A).

In addition, high SDI, middle SDI, low-middle SDI, and low SDI regions exhibited increasing trends in the ASPR for AF/AFL, with the AAPCs being 0.12 (95% CI: 0.07, 0.17), 0.24 (95% CI: 0.21, 0.27), 0.13 (95% CI: 0.12, 0.14), and 0.14 (95% CI: 0.12, 0.17), respectively (Table 1; Figure 5B).

The ASMR for AF/AFL in some SDI regions, including high-middle SDI, low-middle SDI, and low SDI regions, increased from 1990 to 2021, with the AAPCs being 0.1 (95% CI: 0.02, 0.17), 0.92 (95% CI: 0.61, 1.22), and 0.61 (95% CI: 0.14, 1.08), respectively (Table 1; Figure 5C).

Furthermore, the ASDR for AF/AFL in middle SDI, low-middle SDI, and low SDI regions increased from 1990 to 2021, with the AAPCs being 0.11 (95% CI: 0.05, 0.17), 0.53 (95% CI: 0.35, 0.71), and 0.36 (95% CI: 0.12, 0.6), respectively (Table 1; Figure 5D).

### Decomposition analysis

**Figure 6:**
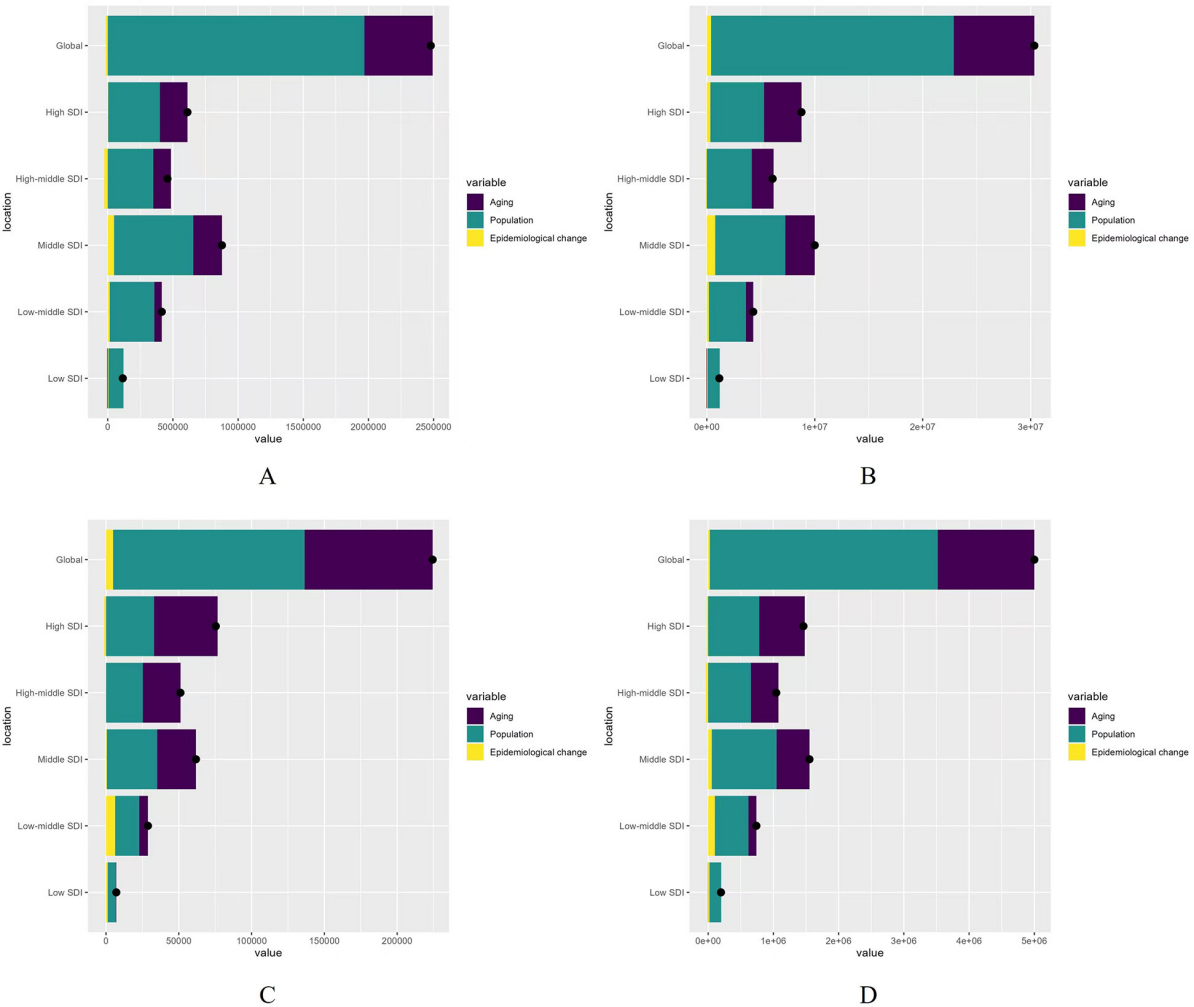
Changes in AF/AFL according to population-level determinants of population growth, aging, and epidemiological change from 1990 to 2021 at the global level and by SDI quintile (the black dot represents the overall value of change contributed by all three components). **(A)** Incidence rate; **(B)** Prevalence rate; **(C)** Mortality rate; **(D)** DALYs rate.

Decomposition analysis was performed to assess the relative contributions of aging, population growth, and epidemiological changes to the global burden of AF/AFL. Over the past 32 years, the global incidence of AF/AFL increased significantly, with the highest rise observed in middle SDI regions. Aging accounted for 21.13% of the worldwide increase in incidence, while population growth contributed 79.45%, predominantly affecting high SDI regions (34.64%) and low SDI regions (99.68%). Epidemiological changes had a slight negative effect (−0.58%) on incidence growth globally, with the most significant negative impact observed in high-middle SDI regions (−5.89%) (Table 2; Figure 6A).

**Table 2:** Changes in burdens of AF/AFL according to population-level determinants and causes from 1990 to 2021.

Location	Measure	Overall difference	Aging	Population	Epidemiological change
Global	Incidence	2,478,355.67	523,703.56 (21.13%)	1,969,014.39 (79.45%)	−14,362.28 (−0.58%)
	Prevalence	30,337,549.95	7,475,653.32 (24.64%)	22,478,372.96 (74.09%)	383,523.67 (1.26%)
	Deaths	224,407.04	88,011.14 (39.22%)	131,569.63 (58.63%)	4,826.27 (2.15%)
	DALYs	5,000,186.12	1,480,687.82 (29.61%)	3,493,677.22 (69.87%)	25,821.08 (0.52%)
High SDI	Incidence	611,751.11	211,890.18 (34.64%)	396,924.74 (64.88%)	2,936.19 (0.48%)
	Prevalence	8,769,393.28	3,474,131.86 (39.62%)	4,973,946.93 (56.72%)	321,314.49 (3.66%)
	Deaths	75,433.58	43,756.99 (58.01%)	32,914.72 (43.63%)	−1,238.13 (−1.64%)
	DALYs	1,461,409.69	697,183.00 (47.71%)	783,925.30 (53.64%)	−19,698.61 (−1.35%)
High-middle SDI	Incidence	457,834.38	136,232.34 (29.76%)	348,568.13 (76.13%)	−26,966.08 (−5.89%)
	Prevalence	6,095,883.09	2,031,686.52 (33.33%)	4,153,178.63 (68.13%)	−88,982.05 (−1.46%)
	Deaths	61,790.67	26,680.31 (43.18%)	34,585.81 (55.97%)	524.55 (0.85%)
	DALYs	1,043,972.51	423,744.49 (40.59%)	654,311.91 (62.68%)	−34,083.89 (−3.26)%
Middle SDI	Incidence	876,355.96	220,523.77 (25.16%)	607,732.42 (69.35%)	48,099.77 (5.49%)
	Prevalence	9,996,830.23	2,727,507.76 (27.28%)	6,500,225.86 (65.02%)	769,096.60 (7.69%)
	Deaths	61,790.67	26,680.31 (43.18%)	34,585.81 (55.97%)	524.55 (0.85%)
	DALYs	1,553,602.51	505,998.34 (32.57%)	992,558.99 (63.89%)	55,045.17 (3.54%)
Low-middle SDI	Incidence	414,807.77	57693.94 (13.91%)	342,431.75 (82.55%)	14,682.08 (3.54%)
	Prevalence	4,297,682.28	689,064.65 (16.03%)	3,421,432.13 (79.61%)	187,185.51 (4.36%)
	Deaths	28,805.08	6,034.53 (20.95%)	16,568.06 (57.52%)	6,202.48 (21.53%)
	DALYs	739,720.39	121,350.68 (16.40%)	516,470.65 (69.82%)	101,899.07 (13.78%)
Low SDI	Incidence	115,752.37	−5,069.40 (−4.38%)	115,380.64 (99.68%)	5,441.14 (4.70%)
	Prevalence	1,153,334.43	−42,627.19 (−3.69%)	1,131,439.74 (98.10%)	64,521.89 (5.59%)
	Deaths	7,055.84	345.07 (4.89%)	5,557.83 (78.77%)	1,152.94 (16.34%)
	DALYs	197,358.17	−3,104.75 (−1.57%)	180,298.30 (91.36%)	20,164.62 (10.22%)

From 1990 to 2021, aging, population growth, and epidemiological changes were the primary drivers of increased prevalence globally, contributing 24.64%, 74.09%, and 1.26%, respectively. Aging contributed most significantly in high SDI regions (39.62%), while population growth had the largest effect on prevalence increase in low SDI regions (98.10%). Epidemiological change accounted for a smaller proportion (1.26%) of the global increase in prevalence, with middle SDI regions experiencing the largest increase in prevalence (7.69%) (Table 2; Figure 6B).

The global increase in deaths from AF/AFL from 1990 to 2021 was driven by aging (39.22%), population growth (58.63%), and epidemiological changes (2.15%). The most significant aging contribution occurred in high SDI regions (58.01%), while population growth had the largest effect on deaths in low SDI regions (78.77%). Epidemiological change had the greatest impact on deaths in low-middle SDI regions (21.53%) (Table 2; Figure 6C).

For DALYs, aging, population growth, and epidemiological changes accounted for 29.61%, 69.87%, and 0.52%, respectively. The largest aging contribution occurred in high SDI regions (47.71%), while the largest effect of population growth on DALYs was seen in low SDI regions (91.36%). Low-middle SDI regions experienced the highest increase in DALYs (13.78%) (Table 2; Figure 6D).

### Changing patterns at different SDI levels and baseline burden

**Figure 7:**
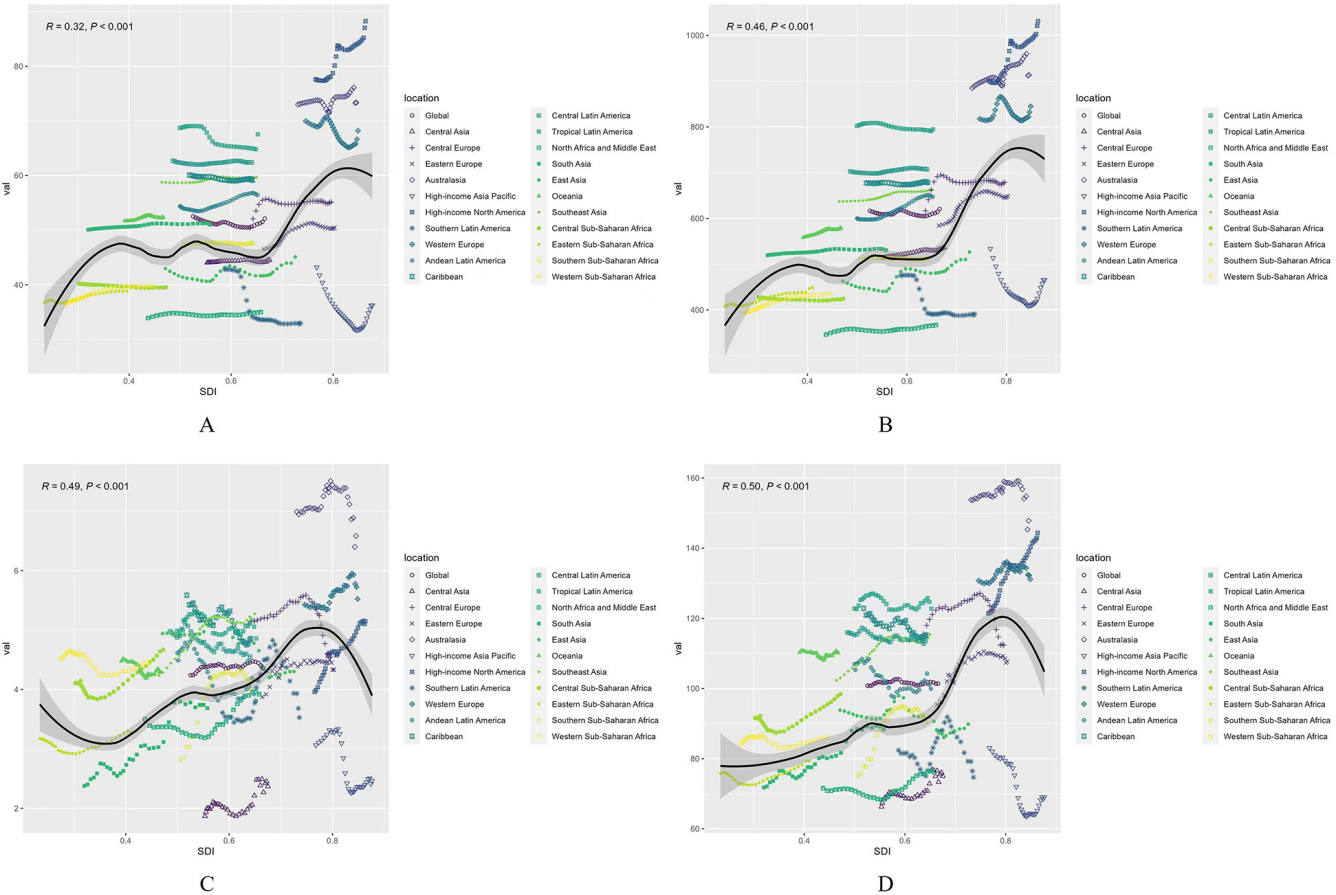
Age-standardized burden rate attributable to AF/AFL across 21 GBD regions by SDI, 1990–2021. **(A)** ASIR; **(B)** ASPR; **(C)** ASMR; **(D)** ASDR. The black line was an adaptive association fitted with adaptive Loess regression based on all data points.

As shown in Figure 7, both the ASIR and ASPR of AF/AFL exhibit a rapid decrease when SDI < 0.4, followed by fluctuations between SDI values of 0.4 and 0.65, and a rapid increase when SDI exceeds 0.65 (Figures 7A,B). The ASMR shows a notable decrease when SDI < 0.4, followed by a sharp increase between SDI values of 0.4 and 0.75, and a subsequent decline when SDI exceeds 0.75 (Figure 7C). The ASDR shows a significant increase when SDI < 0.8, followed by a sharp decrease when SDI exceeds 0.8 (Figure 7D).

### Health inequality in 1990 and 2021

**Figure 8:**
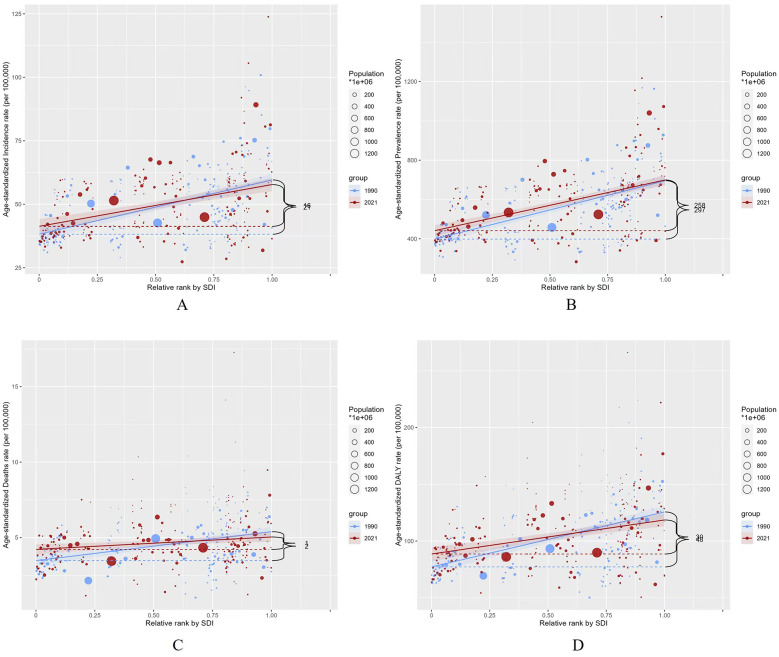
Slope indices inequality for burden of AF/AFL in 1990 and 2021 (the numbers adjacent to the brackets indicate the slopes). **(A)** ASIR; **(B)** ASPR; **(C)** ASMR; **(D)** ASDR.

In both 1990 and 2021, the Social Inequality Index (SII) for incidence was 21 and 16, for prevalence was 297 and 258, for deaths was 2 and 1, and for DALYs was 48 and 30, indicating a positive correlation with the SDI index (Figures 8A–D). These results highlight significant disparities in the burden of AF/AFL between higher and lower SDI nations over the observed period, though the disparity has gradually narrowed.

### Predictions of global burden of AF/AFL from 2022 to 2050

**Figure 9:**
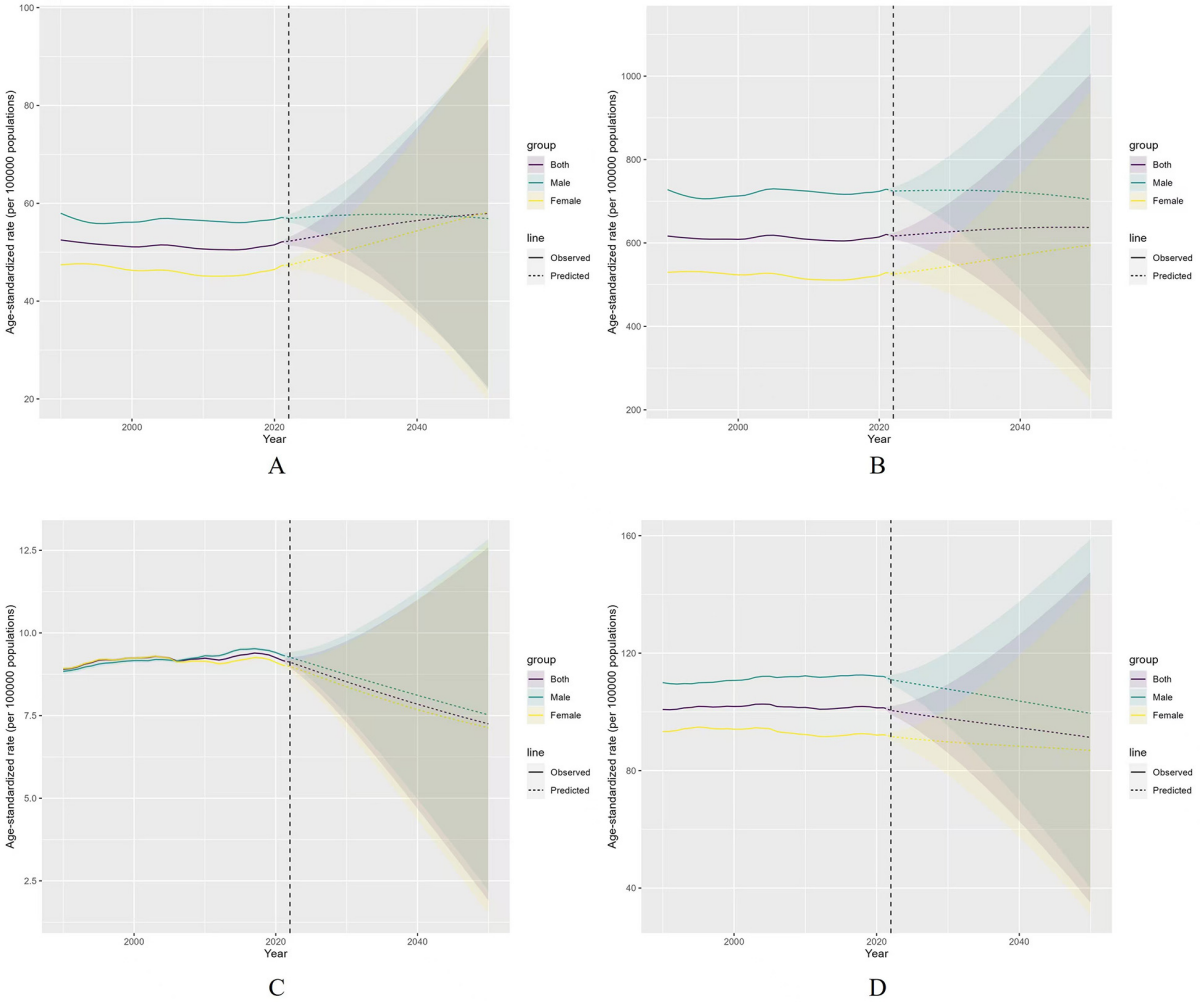
Projected the ASR of AF/AFL for both sexes in 2050. **(A)** ASIR, **(B)** ASPR, **(C)** ASMR, **(D)** ASDR.

Based on the BAPC model predictions, the ASIR of AF/AFL is expected to show an upward trend over the next 29 years. For sex differences, the ASIR is projected to slightly decline for males and increase for females from 2022 to 2050. The ASIR for both sexes is expected to increase from 52.23 to 57.92, with males decreasing from 56.91 to 56.87 and females increasing from 47.36 to 58.19 (Figure 9A).

The ASPR for AF/AFL is projected to remain relatively stable in the future, though it will slightly decline for males and increase markedly for females from 2022 to 2050. The ASPR for both sexes is predicted to rise from 616.50 to 637.06, with males decreasing from 724.35 to 704.23 and females increasing from 533.27 to 965.12 (Figure 9B).

The ASMR for AF/AFL is expected to decrease rapidly for both sexes over the next 29 years globally, from 9.11 to 7.25, with males decreasing from 9.27 to 7.52 and females decreasing from 8.99 to 7.13 (Figure 9C). Similarly, the ASDR for AF/AFL is forecasted to decline for both sexes from 2022 to 2050, with the global rate decreasing from 100.49 to 91.30, males from 110.97 to 99.47, and females from 91.61 to 86.84 (Figure 9D).

## Discussion

Over the past two decades, the burden of AF/AFL has increased annually due to heightened exposure to risk factors, population aging, and prolonged survival of patients (22). Given the escalating burden of AF/AFL, enhancing the understanding and management of its risk factors and promoting early disease prevention have become critical public health priorities. To better assess the recent disease burden of AF/AFL, this study updated the long-term global trends in morbidity and mortality using the latest GBD 2021 data.

Emerging evidence highlights socioeconomic disparities in AF/AFL outcomes. Lower individual income demonstrates significant associations with elevated risks of all-cause mortality and first-onset ischemic stroke. As evidenced by Biancari et al. (10), populations with limited educational attainment and healthcare access experience disproportionately severe AF-related complications. Conversely, studies by Mou et al. (23) reveal lower AF incidence rates among high socioeconomic status cohorts. Nationwide data from China demonstrate a rising AF prevalence, showing positive correlations with SES indicators, female, rural residency, and stroke comorbidity (24).

Our results indicate that the global burden of AF remains substantial. Both the ASIR and ASPR demonstrated an upward trend from 1990 to 2021, while ASMR and ASDR exhibited regional variations. Previous studies have reported a positive correlation between AF/AFL prevalence and the SDI (25). These findings highlight that AF/AFL remains a progressively increasing global challenge. The rising incidence of AF/AFL is primarily attributed to the growing older adult population in developed countries and the increasing prevalence across all age groups in developing nations. Aging has emerged as a significant societal issue in developed countries (26), while developing countries are experiencing a surge in their older adult populations due to their large demographic bases. AF/AFL risk escalates with age (27), and advancements in diagnostic tools have enabled earlier and more accurate detection of AF/AFL, including paroxysmal or asymptomatic cases, contributing to the observed rise in incidence. Besides, there are differences in the diagnosis and treatment of hypertrophic cardiomyopathy between high-income and low-income countries. High-income countries have more resources to research and implement advanced diagnostic and therapeutic methods. In contrast, low-income countries face challenges in accessing diagnostic equipment, trained personnel, and affordable medications, leading to reduced quality of life and lower life expectancy for affected individuals (10, 28).

Regarding mortality, our findings reveal pronounced regional disparities, closely linked to SDI. In high-SDI regions, mortality rates have fluctuated within a narrow range in recent years, whereas low-SDI regions have witnessed a marked upward trend. This disparity can be attributed to differences in health awareness and access to care. For instance, the REGARDS study reported that AF awareness among U.S. residents aged ≥45 years was 60% in 2003–2007 (29), while a 2013 community survey in Ireland found an awareness rate of 61.9% among residents aged ≥50 years (30). These findings underscore that low AF/AFL awareness is a global issue, likely exacerbated in low-SDI regions, where limited awareness may increase the risk of complications and mortality. Additionally, discrepancies in therapeutic strategies, including the use of novel oral anticoagulants, left atrial appendage closure, and catheter or cryoballoon ablation, vary across regions based on economic development, further influencing outcomes.

This study also highlights gender disparities in the AF/AFL disease burden. In 2021, males exhibited a higher incidence of AF/AFL than females, while females experienced higher mortality and DALY rates. This may reflect the global male-to-female population ratio, with males constituting a larger demographic base. Additionally, estrogen has been shown to exert a protective effect against AF/AFL by prolonging atrial refractoriness, potentially explaining the increased incidence among postmenopausal women (31). Furthermore, income disparities between genders may limit women's access to healthcare resources (13). Treatment strategies for AF/AFL also differ by gender, with female patients less likely to receive rhythm control interventions (32, 33). These findings underscore necessitating tailored strategies to optimize clinical outcomes.

The prevention strategies for atrial AF/AFL are profoundly reshaping cardiovascular disease management through multidimensional interventions, with growing emphasis on the synergistic role of dietary modifications and early screening initiatives. Evidence confirms that reduced alcohol/caffeine intake and adoption of Mediterranean-style diets mitigate atrial electrical remodeling (34, 35). Concurrently, early detection programs leveraging wearable technology and community healthcare networks have advanced diagnostic timelines by 2–3 years through identification of subclinical arrhythmias and high-risk populations, creating critical opportunities for timely anticoagulation and risk factor management (36).

Projections from the Bayesian Age-Period-Cohort (BAPC) models suggest that ASIR and ASPR will fluctuate within a narrow range over the next two decades, while ASMR and ASDR are expected to decline gradually by 2050. These trends reflect the effectiveness of strengthened healthcare systems, scaled-up interventions, efforts to address within-country disparities, and comprehensive actions targeting social determinants of health. However, while the burden of AF/AFL is anticipated to decrease, it remains imperative to minimize disparities between high- and low-SDI regions through international collaboration and region-specific public health strategies.

Building on the findings, multisectoral policy interventions are urgently required to address the escalating burden of AF/AFL. First, early detection systems should be prioritized, particularly in high-SDI regions with aging populations, by integrating routine AF/AFL screening into cardiovascular risk assessments for older adults. Expanding access to portable ECG monitors could enhance case identification, especially for asymptomatic or paroxysmal episodes. Second, equitable resource allocation must be strengthened through global partnerships to support low-SDI regions in establishing standardized anticoagulation protocols, catheter ablation infrastructure, and stroke prevention programs, thereby mitigating care disparities. Gender-sensitive strategies are critical to address underdiagnosis and undertreatment in women, including public health campaigns to raise awareness and clinical guidelines tailored to sex-specific risk profiles. Third, integrated care models should be promoted to bridge primary and tertiary healthcare systems, leveraging telemedicine for timely specialist consultations in underserved areas. Concurrently, population-level measures—such as taxation on tobacco/alcohol, sodium reduction initiatives, and obesity prevention programs—are essential to curb modifiable risk factors. Finally, establishing a global AF/AFL registry would enable real-time monitoring of epidemiological trends, therapeutic outcomes, and policy impacts, fostering evidence-based adjustments to intervention frameworks. These steps, aligned with the WHO's Sustainable Development Goals, could substantially reduce the global burden while advancing health equity.

There remain some limitations in this study. First, it relies on secondary data from the GBD study, which may compromise data quality. Second, underdiagnosis and misdiagnosis in less developed countries may lead to an underestimation of the disease burden. Third, projections of future trends are based on current conditions and may not account for unforeseen changes. Several potential confounding factors warrant consideration. Geographical disparities in healthcare access may influence screening participation rates and treatment adherence, particularly affecting underserved populations. Additionally, temporal variations in diagnostic criteria for atrial arrhythmias (e.g., evolving ECG interpretation guidelines or device-detected AF definitions) could introduce heterogeneity in case identification across study periods.

## Conclusion

AF/AFL persists as a critical global health burden, with escalating incidence and prevalence fueled by aging populations and enhanced detection methods. Mitigating this burden requires targeted mitigation of healthcare disparities arising from socioeconomic gradients through equitable resource distribution.

## Data Availability

Publicly available datasets were analyzed in this study. This data can be found here: http://www.healthdata.org.
