# Supplementary material for: Global burden and health inequality of atrial fibrillation/atrial flutter from 1990 to 2021
Source: Front Cardiovasc Med. 2025 May 21;12:1585980. doi: 10.3389/fcvm.2025.1585980 (PMC12133759; doi:10.3389/fcvm.2025.1585980)
Supplement: Supplementary file 1 [file Table1.pdf]

Supplementary Table 1 Global burden of AF/AFL in 2021.

location	Incidence		Prevalence		Deaths		DALYs	
	Number (95% UI)	ASIR (95% UI, per 100,000)	Number (95% UI)	ASPR (95% UI, per 100,000)	Number (95% UI)	ASMR (95% UI, per 100,000)	Number (95% UI)	ASDR (95% UI, per 100,000)
Global	4484926 (3610620-5706019)	52.12 (41.85-66.23)	52552045 (43137876-64963854)	620.51 (511.36-768.88)	338947 (288954-368613)	4.36 (3.69-4.75)	8358894 (6970688-10133489)	101.4 (84.89-122.41)
**SDI regions**								
High SDI	1334730 (1139377-1572638)	65.1 (56.11-76.05)	17401068 (15107026-20202156)	788.35 (690.97-910.9)	125622 (103230-137669)	4.66 (3.88-5.08)	2788952 (2345598-3317970)	118.88 (99.51-141.23)
High-middle SDI	934174 (740417-1198977)	47.16 (37.67-60.25)	11539353 (9330859-14532463)	581.39 (473.47-731.22)	79214 (67067-88012)	4.29 (3.62-4.77)	1871378 (1545511-2273392)	96.58 (79.91-116.81)
Middle SDI	1330376 (1031639-1752211)	51.11 (39.2-67.85)	14670503 (11595532-19210983)	579.06 (457.58-748.76)	83853 (70992-94818)	4.26 (3.57-4.83)	2256889 (1828561-2776292)	96.28 (79.25-117.4)
Low-middle SDI	682885 (523053-91203	50.99 (38.44-67.86	6929826 (5413491-903356	546.49 (425.68-711.16	39263 (32069-46278	4.08	1110930 (893165-138576	94.25 (76.45-116.7

location	Incidence		Prevalence		Deaths		DALYs	
	Number (95% UI)	ASIR (95% UI, per 100,000)	Number (95% UI)	ASPR (95% UI, per 100,000)	Number (95% UI)	ASMR (95% UI, per 100,000)	Number (95% UI)	ASDR (95% UI, per 100,000)
	6)	)	2)	)	)	(3.34-4.81)	4)	1)
Low SDI	198494 (153240-261939)	43.25 (32.7-57.75)	1959344 (1535182-2587655)	463.23 (362.02-602.71)	10623 (7284-13876)	3.74 (2.57-4.9)	321963 (242299-416819)	84.52 (63.65-108.24)
**GBD super regions**								
Central Europe, Eastern Europe, and Central Asia	335651 (259179-433645)	51.13 (39.86-65.65)	4305271 (3406712-5444005)	647.85 (514.47-815.74)	28187 (25436-29980)	4.25 (3.83-4.52)	707096 (587078-854537)	106.32 (88.39-128.83)
High-income	1406726 (1201489-1671800)	66.35 (57.33-77.61)	18539352 (16001529-21465804)	797.66 (699.07-918.3)	135998 (110821-149407)	4.64 (3.83-5.06)	2976201 (2503741-3543199)	119.23 (99.47-141.93)
Latin America and Caribbean	392071 (303509-516388)	63.64 (48.95-84.38)	4454916 (3518285-5762071)	735.11 (577.41-948.28)	26704 (22895-29122)	4.64 (3.98-5.06)	693667 (564426-849785)	116.22 (94.78-142.17)
North Africa and	145164	35.04	1417367	366.92	11182	3.92	265649	76.1

location	Incidence		Prevalence		Deaths		DALYs	
	Number (95% UI)	ASIR (95% UI, per 100,000)	Number (95% UI)	ASPR (95% UI, per 100,000)	Number (95% UI)	ASMR (95% UI, per 100,000)	Number (95% UI)	ASDR (95% UI, per 100,000)
Middle East	(114585-18608 6)	(26.72-45.54)	(1140557-178891 1)	(291.62-468.4)	(9280-12594)	(3.19-4.44)	(218559-309833)	(63.33-88.63)
South Asia	698920 (527866-93481 8)	51.01 (38.09-67.89)	6882583 (5295961-902613 9)	530.9 (410.19-696.69)	36165 (27041-46073)	3.69 (2.75-4.71)	1071260 (820127-136668 0)	88.3 (68.02-111.4 5)
Southeast Asia, East Asia, and Oceania	1326901 (1024793-1737 391)	48.18 (37.23-63.8)	15148767 (12007256-19710 445)	553.99 (441.79-720.56)	90253 (74089-10515 8)	4.49 (3.68-5.29)	2343043 (1885946-29158 36)	95.17 (78.28-115.4 8)
Sub-Saharan Africa	179494 (140395-23337 9)	40.75 (31.09-54.56)	1803788 (1418756-235982 9)	449.09 (353.18-585.76)	10458 (8229-12861)	4.05 (3.18-4.9)	301978 (233263-382254)	85.9 (67.68-106.6 5)
**GBD regions**								
Central Asia	36231 (27486-47288)	44.53 (33.54-57.85)	392829 (305783-509896)	536.72 (410.76-698.74)	1386 (1241-1519)	2.37 (2.09-2.6)	52600 (41314-67337)	75 (59.9-95.5)

location	Incidence		Prevalence		Deaths		DALYs	
	Number (95% UI)	ASIR (95% UI, per 100,000)	Number (95% UI)	ASPR (95% UI, per 100,000)	Number (95% UI)	ASMR (95% UI, per 100,000)	Number (95% UI)	ASDR (95% UI, per 100,000)
Central Europe	122626 (94472-154039)	55.16 (44.12-68.47)	1585129 (1281858-1949452)	679 (554.31-827.23)	11104 (9859-11914)	4.55 (4.03-4.88)	265917 (225064-316547)	112.31 (94.56-134.27)
Eastern Europe	176794 (133418-231758)	50.47 (38.83-65.64)	2327313 (1805306-3029889)	648.92 (507-836.25)	15697 (14042-17056)	4.33 (3.87-4.71)	388580 (318353-470379)	107.73 (88.25-130.33)
Australasia	38348 (29260-50108)	73.34 (57.3-94.35)	508534 (400838-650591)	913.63 (725.73-1163.47)	4337 (3529-4789)	6.58 (5.39-7.25)	87583 (72120-105463)	147.83 (121.51-179.59)
High-income Asia Pacific	152471 (116375-199323)	36.27 (29.16-46.41)	2165450 (1737905-2727227)	465.22 (383.84-577.04)	17112 (13415-19331)	2.46 (2.01-2.74)	364512 (301067-443767)	69.03 (56.4-84.74)
High-income North America	586320 (534188-649281)	88.24 (80.92-97.25)	7106415 (6544296-7755026)	1031.17 (952.3-1117.88)	39066 (32116-42759)	5.15 (4.27-5.61)	1015978 (854240-1202900)	144.37 (121-171.39)
Southern Latin	28852	33	348813	391.08	3299	3.53	67844	74.67

location	Incidence		Prevalence		Deaths		DALYs	
	Number (95% UI)	ASIR (95% UI, per 100,000)	Number (95% UI)	ASPR (95% UI, per 100,000)	Number (95% UI)	ASMR (95% UI, per 100,000)	Number (95% UI)	ASDR (95% UI, per 100,000)
America	(23283-35802)	(26.86-40.72)	(291741-425439)	(328.22-476.31)	(2853-3562)	(3.06-3.81)	(58167-78537)	(63.84-86.8)
Western Europe	600735 (491661-740671)	68.19 (56.6-82.27)	8410140 (7063579-10013004)	844.93 (717.15-992.46)	72184 (58846-79292)	5.52 (4.56-6.04)	1440284 (1208224-1709735)	131.15 (108.91-156.67)
Andean Latin America	33004 (25562-43750)	56.44 (43.34-75.08)	371258 (290314-478820)	647.36 (505.19-835.55)	2079 (1699-2494)	3.81 (3.11-4.57)	56583 (44608-69532)	99.93 (78.68-122.31)
Caribbean	31921 (24658-42125)	59.14 (45.45-78.13)	367640 (289060-476682)	678.59 (533.41-881.12)	2731 (2349-3048)	4.86 (4.2-5.42)	63053 (51870-76838)	115.24 (94.57-140.74)
Central Latin America	154316 (119073-205022)	62.38 (47.92-83.05)	1711299 (1340757-2226156)	707.02 (552.56-921.13)	10354 (8980-11386)	4.55 (3.95-5.01)	269931 (218012-327388)	113.57 (91.93-137.77)
Tropical Latin America	172830 (133612-22436	67.55 (52.09-88.35	2004719 (1580577-259990	794.78 (625.65-1025.1	11540 (9640-12679)	4.86 (4.05-5.34)	304099 (245416-375972)	122.75 (99.62-151.6

location	Incidence		Prevalence		Deaths		DALYs	
	Number (95% UI)	ASIR (95% UI, per 100,000)	Number (95% UI)	ASPR (95% UI, per 100,000)	Number (95% UI)	ASMR (95% UI, per 100,000)	Number (95% UI)	ASDR (95% UI, per 100,000)
	9)	)	5)	8)				5)
North Africa and Middle East								
South Asia								
East Asia	953898 (737314-1249815)	45.11 (35.12-59.61)	11215165 (8885909-14572495)	526.44 (420.6-683.56)	67666 (54232-80814)	4.3 (3.42-5.18)	1723468 (1364785-2143575)	89.83 (72.42-109.59)
Oceania	3484 (2741-4545)	52.33 (39.94-69.52)	34994 (27519-45179)	578.44 (455.22-750.19)	185 (132-238)	4.29 (3.16-5.46)	6606 (5044-8584)	110.15 (86.19-141.7)
Southeast Asia	369518 (285921-483602)	59.66 (45.6-79.28)	3898608 (3091004-5118881)	662.33 (519.61-854.36)	22401 (18974-25759)	5.27 (4.39-6.12)	612969 (501403-745497)	115.4 (95.88-139.11)
Central Sub-Saharan	19101 (14928-25118)	39.56 (29.91-52.82	182594 (142529-241993)	424.57 (331.96-559.66	1311 (886-1982)	4.67 (3.16-7.09)	37999 (27202-52014)	98.45 (70.85-138.1

location	Incidence		Prevalence		Deaths		DALYs	
	Number (95% UI)	ASIR (95% UI, per 100,000)	Number (95% UI)	ASPR (95% UI, per 100,000)	Number (95% UI)	ASMR (95% UI, per 100,000)	Number (95% UI)	ASDR (95% UI, per 100,000)
Africa		)		)				8)
Eastern Sub-Saharan Africa	62823 (49401-81796)	39.66 (30.44-52.91)	645690 (507531-839253)	448.76 (354.06-589.55)	3166 (1927-4731)	3.28 (1.97-4.89)	103373 (74839-140257)	79.25 (57.05-108.27)
Southern Sub-Saharan Africa	25556 (19614-33834)	47.6 (36.27-63.27)	258590 (201941-342437)	512.78 (398.19-671.03)	1413 (1228-1551)	4 (3.39-4.44)	41787 (34114-50581)	90.4 (74.56-108.99)
Western Sub-Saharan Africa	72014 (56373-94024)	39.82 (30.36-53.28)	716915 (561805-945355)	434.84 (339.54-567.05)	4568 (3662-5235)	4.46 (3.65-5.13)	118819 (92666-146393)	86.12 (68.4-104.09)
**Countries/territories**								
Afghanistan	2735 (2056-3602)	33.33 (24.48-44.48)	25639 (19701-33184)	338.76 (257.55-442.8)	179 (111-264)	3.28 (2.04-4.86)	5074 (3663-6761)	72.08 (51.55-95.15)

location	Incidence		Prevalence		Deaths		DALYs	
	Number (95% UI)	ASIR (95% UI, per 100,000)	Number (95% UI)	ASPR (95% UI, per 100,000)	Number (95% UI)	ASMR (95% UI, per 100,000)	Number (95% UI)	ASDR (95% UI, per 100,000)
Albania	2070 (1507-2764)	46.19 (34.59-60.4)	26254 (20002-34169)	582.74 (445.93-753.86)	181 (132-229)	4.72 (3.41-6.04)	4399 (3431-5468)	103.15 (81.18-126.41)
Algeria	11749 (8706-15697)	35.37 (25.91-47.68)	116519 (89354-153163)	376.73 (287.03-499.12)	1114 (824-1390)	5.97 (4.5-7.24)	23845 (18698-29189)	95.93 (76.67-116.45)
American Samoa	27 (21-36)	59.56 (45.61-79.26)	289 (229-380)	672.65 (528.47-875.08)	2 (1-3)	6.46 (4.61-8.54)	58 (45-71)	143.68 (111.47-176.76)
Andorra	91 (68-119)	60.36 (45.23-78.41)	1172 (905-1510)	743.99 (574.87-956.34)	7 (5-9)	3.66 (2.64-4.82)	170 (130-217)	102.38 (77.85-131.72)
Angola	4344 (3391-5660)	41.84 (31.79-55.87)	41399 (32326-54332)	452.08 (354.11-588.63)	279 (194-392)	4.86 (3.35-6.83)	8368 (6207-11093)	102.72 (76.04-135.77)
Antigua and	60	57.46	664	657.2	5	6.61	121	132.09

location	Incidence		Prevalence		Deaths		DALYs	
	Number (95% UI)	ASIR (95% UI, per 100,000)	Number (95% UI)	ASPR (95% UI, per 100,000)	Number (95% UI)	ASMR (95% UI, per 100,000)	Number (95% UI)	ASDR (95% UI, per 100,000)
Barbuda	(46-80)	(43.94-75.87)	(518-869)	(512.46-858.3)	(5-6)	(5.97-7.14)	(101-147)	(111.79-158.06)
Argentina	16008 (13581-19205)	28.5 (24.36-34.01)	194835 (169094-228621)	340.72 (296.16-399.78)	2027 (1752-2197)	3.42 (2.95-3.7)	40198 (34929-46587)	69.17 (59.96-80.23)
Armenia	2013 (1479-2662)	45.65 (34.23-60.63)	24452 (18788-32257)	556.65 (424.75-729.78)	108 (93-122)	2.53 (2.18-2.86)	3355 (2607-4231)	76.87 (59.76-96.05)
Australia	32531 (24219-42746)	74.05 (56.85-96.39)	435826 (338605-563002)	928.31 (725.71-1201.33)	3608 (2927-3990)	6.42 (5.24-7.08)	73571 (60143-89381)	146.76 (119.81-179.53)
Austria	18808 (17782-19775)	105.58 (100.49-110.74)	241015 (230229-251784)	1217.22 (1164.84-1272.17)	1651 (1349-1818)	6.75 (5.58-7.41)	37096 (30927-43902)	175.92 (146.38-208.58)
Azerbaijan	4617 (3523-6113)	44.9 (33.78-58.97	48767 (38083-64570)	540.88 (415.38-701.51	124 (93-154)	1.79 (1.39-2.21)	5902 (4361-7951)	67.92 (51.38-90.14

location	Incidence		Prevalence		Deaths		DALYs	
	Number (95% UI)	ASIR (95% UI, per 100,000)	Number (95% UI)	ASPR (95% UI, per 100,000)	Number (95% UI)	ASMR (95% UI, per 100,000)	Number (95% UI)	ASDR (95% UI, per 100,000)
Bahamas	234 (181-309)	59.29 (45.09-78.28)	2533 (1987-3335)	674.57 (529.29-883.91)	19 (16-22)	6.06 (5.1-7.02)	469 (380-561)	132.64 (108.37-157.69)
Bahrain	297 (229-382)	36.29 (26.61-48.17)	2667 (2055-3468)	386.71 (293.82-505.55)	15 (8-20)	5.66 (2.58-7.9)	442 (316-569)	94.91 (57.64-122.79)
Bangladesh	60982 (45614-80831)	46.19 (34.26-61.31)	623060 (480696-812961)	494.7 (380.67-643.93)	4552 (3252-6400)	5 (3.64-6.99)	109146 (82747-147679)	97.3 (74.5-129.97)
Barbados	316 (237-425)	60.95 (46.79-81.94)	3693 (2829-4833)	701.8 (540.68-913.61)	29 (24-34)	5.74 (4.81-6.66)	641 (513-774)	123.06 (98.96-148.54)
Belarus	7544 (5606-9888)	47.24 (35.87-61.53)	101036 (78621-132534)	620.57 (486.13-809.95)	677 (566-781)	4.06 (3.41-4.69)	16740 (13493-20892)	101.79 (82.13-126.55)

location	Incidence		Prevalence		Deaths		DALYs	
	Number (95% UI)	ASIR (95% UI, per 100,000)	Number (95% UI)	ASPR (95% UI, per 100,000)	Number (95% UI)	ASMR (95% UI, per 100,000)	Number (95% UI)	ASDR (95% UI, per 100,000)
Belgium	11602 (8964-15260)	53.01 (41.85-67.78)	163236 (131345-207711)	675.51 (553.96-847.21)	1396 (1088-1557)	4.21 (3.35-4.67)	27975 (22943-33735)	103.49 (84.22-126.61)
Belize	175 (136-229)	60.74 (45.89-80.02)	1881 (1485-2467)	693.37 (538.53-897.5)	11 (9-12)	4.61 (3.97-5.16)	297 (239-365)	113.27 (91.6-138.44)
Benin	1743 (1366-2273)	36.28 (27.75-48.29)	17562 (13735-23147)	396.48 (308.75-520.98)	108 (82-137)	3.66 (2.75-4.62)	2817 (2218-3600)	73.63 (57.93-92.8)
Bermuda	82 (62-110)	60.11 (45.87-79.58)	1007 (782-1312)	700.19 (547.46-909.57)	7 (6-9)	4.45 (3.69-5.36)	160 (130-197)	105.58 (84.94-130.79)
Bhutan	269 (202-360)	46.27 (34.64-61.83)	2770 (2170-3630)	492.91 (384.1-647.49)	22 (15-30)	4.47 (3.09-6.28)	503 (379-649)	93.85 (70.8-121.02)
Bolivia	4880	55.4	51917	622.3	358	5.65	9440	123.22

location	Incidence		Prevalence		Deaths		DALYs	
	Number (95% UI)	ASIR (95% UI, per 100,000)	Number (95% UI)	ASPR (95% UI, per 100,000)	Number (95% UI)	ASMR (95% UI, per 100,000)	Number (95% UI)	ASDR (95% UI, per 100,000)
(Plurinational State of)	(3734-6425)	(42.03-74.02)	(40535-67894)	(488.06-809.65)	(258-486)	(4.13-7.54)	(7098-11874)	(93.91-154.0 8)
Bosnia and Herzegovina	2895 (2119-3855)	46.03 (34.49-60.17)	36920 (28206-47883)	574.34 (442.67-741.06)	258 (207-309)	4.07 (3.26-4.87)	6410 (5131-7828)	99.83 (80.18-121.3 5)
Botswana	581 (449-758)	42.95 (32.63-57.14)	5721 (4459-7586)	467.19 (359.37-610.96)	26 (20-36)	3.24 (2.42-4.69)	862 (665-1099)	77.73 (60.71-99.9)
Brazil	169133 (130789-21970 3)	67.63 (52.17-88.49)	1961680 (1546310-254433 2)	795.32 (626.23-1025.7 1)	11250 (9412-12337)	4.84 (4.04-5.31)	297032 (239336-367908)	122.58 (99.23-151.6 4)
Brunei Darussalam	130 (101-166)	35.52 (27.3-45.61)	1479 (1155-1910)	452.03 (354.84-582.96)	12 (10-13)	6.22 (5.18-7.43)	328 (271-388)	122.03 (102.46-143. 98)
Bulgaria	6484 (4590-8604)	45.66 (33.96-60.04	86405 (64937-113573)	564.76 (430.67-735.55	902 (786-1026)	6.17 (5.4-6.98)	19898 (16801-23531)	132.45 (112.78-156.

location	Incidence		Prevalence		Deaths		DALYs	
	Number (95% UI)	ASIR (95% UI, per 100,000)	Number (95% UI)	ASPR (95% UI, per 100,000)	Number (95% UI)	ASMR (95% UI, per 100,000)	Number (95% UI)	ASDR (95% UI, per 100,000)
		)		)				09)
Burkina Faso	3131 (2428-4105)	36.46 (27.89-48.51)	31626 (24731-41910)	397.21 (311.38-519)	270 (181-365)	5.13 (3.49-6.87)	6312 (4577-8276)	93.72 (69-121.53)
Burundi	1690 (1320-2214)	37.32 (28.58-49.92)	17035 (13278-22301)	418.87 (326.88-552.33)	70 (34-135)	2.74 (1.27-5.53)	2555 (1694-3792)	69.88 (45.58-107.87)
Cabo Verde	163 (127-214)	37.51 (28.45-50.47)	1754 (1380-2302)	419.74 (323.1-551.73)	22 (16-28)	5.43 (3.85-6.88)	387 (300-476)	93.89 (72.94-115.1)
Cambodia	5973 (4647-7812)	52.94 (40.15-69.72)	61483 (48621-81182)	578.56 (457.17-751.32)	279 (217-359)	4.3 (3.27-5.61)	9138 (7031-11349)	98.48 (76.53-122.26)
Cameroon	4541 (3527-5945)	39.23 (29.67-51.86)	45126 (35031-58573)	432.9 (335.41-563.68)	324 (245-422)	5.21 (4-6.66)	8308 (6392-10485)	96.96 (75.89-120.43)

location	Incidence		Prevalence		Deaths		DALYs	
	Number (95% UI)	ASIR (95% UI, per 100,000)	Number (95% UI)	ASPR (95% UI, per 100,000)	Number (95% UI)	ASMR (95% UI, per 100,000)	Number (95% UI)	ASDR (95% UI, per 100,000)
Canada	58043 (42013-77003)	80.6 (59.82-106.65)	731819 (554975-954038)	958.83 (733.17-1244.27)	3777 (3164-4143)	4.29 (3.62-4.69)	99213 (77674-125422)	125.65 (97.53-160.69)
Central African Republic	737 (569-976)	39.69 (30.18-52.71)	6761 (5202-8922)	416.26 (325.14-546.14)	41 (24-63)	4.24 (2.44-6.54)	1396 (964-1962)	94.52 (64.64-133.89)
Chad	1862 (1437-2449)	35.4 (26.67-47.34)	18475 (14434-24478)	383.73 (298.18-504.47)	113 (77-151)	3.82 (2.62-5.07)	3120 (2274-4079)	76.68 (56.2-97.97)
Chile	10713 (8108-14239)	41.63 (31.85-55.05)	127594 (98729-165412)	489.14 (379.61-632.16)	995 (851-1075)	3.73 (3.19-4.02)	22386 (18415-26886)	85.2 (70.05-102.61)
China	916180 (707384-1201381)	44.92 (34.96-59.42)	10775721 (8531627-14014036)	524 (418.15-681.23)	64728 (51765-77729)	4.33 (3.43-5.23)	1653117 (1303681-2056459)	89.76 (72.13-109.67)
Colombia	31451	56.71	361800	650.75	2331	4.03	57513	102.82

location	Incidence		Prevalence		Deaths		DALYs	
	Number (95% UI)	ASIR (95% UI, per 100,000)	Number (95% UI)	ASPR (95% UI, per 100,000)	Number (95% UI)	ASMR (95% UI, per 100,000)	Number (95% UI)	ASDR (95% UI, per 100,000)
	(24378-41668)	(43.35-75.87)	(282565-475575)	(507.97-853.91)	(1893-2705)	(3.31-4.67)	(46273-72041)	(82.58-129.17)
Comoros	170 (132-226)	36.7 (28.01-49.08)	1811 (1415-2366)	415.57 (326.17-548.36)	10 (6-17)	3.34 (1.79-5.53)	303 (209-425)	76.29 (51.65-110.47)
Congo	1025 (797-1332)	42.47 (32.26-56.38)	9845 (7730-12833)	462.37 (360.8-600.06)	80 (56-101)	5.97 (4.33-7.41)	2231 (1708-2802)	118.63 (92.06-145.96)
Cook Islands	15 (12-20)	59.44 (45.14-78.03)	178 (138-232)	683.51 (533.18-886.86)	1 (1-2)	5.99 (4.2-7.74)	33 (25-43)	133.97 (101.01-170.52)
Costa Rica	3442 (2653-4536)	62.53 (48-82.88)	39959 (31002-51910)	727.25 (561.47-946.4)	250 (207-282)	4.3 (3.59-4.82)	6111 (4936-7505)	109.7 (88.42-135.24)
Croatia	3045 (2682-3466)	34.78 (30.93-39.18	39620 (35576-44450)	420.02 (381.35-470.13	363 (319-401)	3.51 (3.09-3.87)	7519 (6521-8661)	76.37 (65.94-88.41

location	Incidence		Prevalence		Deaths		DALYs	
	Number (95% UI)	ASIR (95% UI, per 100,000)	Number (95% UI)	ASPR (95% UI, per 100,000)	Number (95% UI)	ASMR (95% UI, per 100,000)	Number (95% UI)	ASDR (95% UI, per 100,000)
		)		)				)
Cuba	11429 (8751-15178)	58.1 (44.65-76.07)	136060 (106604-175458)	671.42 (527.93-869.79)	1090 (943-1220)	4.87 (4.22-5.46)	23660 (19350-29217)	113.08 (92.16-140.13)
Cyprus	928 (720-1218)	42.95 (34.02-55.26)	10427 (8434-13170)	487.17 (395.53-615.62)	123 (102-143)	8.36 (6.79-9.85)	2333 (1967-2795)	129.79 (108.76-154.1)
Czechia	17905 (14041-20271)	81.97 (65.98-91.63)	228258 (188006-254737)	981.52 (815.26-1089.62)	1123 (973-1237)	4.68 (4.06-5.15)	31633 (25775-37952)	135.11 (109.98-161.89)
Côte d'Ivoire	3940 (3081-5145)	38.46 (29.28-51.82)	38549 (30111-50565)	424.45 (328.78-553.75)	244 (188-307)	4.71 (3.72-5.83)	6579 (5111-8188)	89.33 (71.39-107.83)
Democratic People's Republic of	15152 (11772-20144)	48.09 (37.23-63.29)	166218 (132256-214516)	532.4 (422.66-687.42)	1167 (886-1665)	5.09 (3.77-7.73)	29340 (22444-38029)	103.98 (80.31-138.02)

location	Incidence		Prevalence		Deaths		DALYs	
	Number (95% UI)	ASIR (95% UI, per 100,000)	Number (95% UI)	ASPR (95% UI, per 100,000)	Number (95% UI)	ASMR (95% UI, per 100,000)	Number (95% UI)	ASDR (95% UI, per 100,000)
Korea								
Democratic Republic of the Congo	12383 (9617-16381)	38.47 (28.94-51.58)	118526 (92188-158139)	411.45 (320.33-546.46)	854 (527-1390)	4.45 (2.78-7.34)	24604 (16933-35653)	94.61 (66.05-140.02)
Denmark	7912 (5907-10323)	70.03 (54.45-89.03)	111718 (88772-138463)	908.22 (736.43-1119.51)	874 (741-955)	6.06 (5.17-6.6)	18911 (15746-22897)	145.11 (120.42-176.86)
Djibouti	224 (175-293)	37.38 (28.63-49.61)	2213 (1710-2891)	424.62 (335.81-555.29)	10 (7-15)	3.79 (2.49-5.46)	368 (267-489)	83.68 (61.71-110.9)
Dominica	49 (37-65)	59.66 (45.28-78.68)	544 (418-711)	668.86 (514.54-861.99)	5 (5-6)	7.79 (6.56-9.08)	115 (95-138)	150.75 (124.68-179.75)
Dominican Republic	5823 (4490-7736)	58.97 (45.15-78.8)	64889 (50825-84248)	667.32 (520.85-869.63)	464 (361-587)	4.96 (3.88-6.24)	11054 (8859-13676)	114.8 (92.06-141.9)

location	Incidence		Prevalence		Deaths		DALYs	
	Number (95% UI)	ASIR (95% UI, per 100,000)	Number (95% UI)	ASPR (95% UI, per 100,000)	Number (95% UI)	ASMR (95% UI, per 100,000)	Number (95% UI)	ASDR (95% UI, per 100,000)
Ecuador	8791 (6715-11724)	54.15 (41.24-71.88)	98459 (77061-128281)	615.7 (480.28-800.86)	553 (464-656)	4.01 (3.4-4.71)	14843 (11786-18496)	96.93 (77.68-119.73)
Egypt	19786 (14849-25927)	36.92 (27.14-49.42)	184697 (143378-239672)	393 (297.72-514.22)	971 (799-1145)	3.61 (2.87-4.25)	30353 (24425-37293)	75.86 (62.38-91.21)
El Salvador	3647 (2778-4768)	58.15 (44.23-76.71)	42585 (33215-55380)	665.9 (520.53-865.31)	402 (307-494)	5.36 (4.13-6.56)	8071 (6523-9719)	119.8 (96.6-144.65)
Equatorial Guinea	205 (159-266)	44.12 (33.56-58.64)	1997 (1582-2592)	487.69 (375.94-633.88)	15 (10-21)	5.43 (3.59-7.37)	407 (299-528)	111.1 (83.53-142.52)
Eritrea	883 (684-1162)	35.13 (26.99-47.06)	8704 (6755-11290)	391.78 (304.41-516.89)	49 (26-86)	3.94 (2.07-7.07)	1612 (1063-2379)	84.53 (54.92-128.65)
Estonia	1149	46.91	17269	619.55	190	5.33	3559	114.15

location	Incidence		Prevalence		Deaths		DALYs	
	Number (95% UI)	ASIR (95% UI, per 100,000)	Number (95% UI)	ASPR (95% UI, per 100,000)	Number (95% UI)	ASMR (95% UI, per 100,000)	Number (95% UI)	ASDR (95% UI, per 100,000)
	(859-1498)	(35.52-60.22)	(13159-22327)	(480.95-800.04)	(161-212)	(4.54-5.96)	(3013-4202)	(94.72-136.36)
Eswatini	217 (167-287)	43.34 (32.97-57.62)	2108 (1632-2790)	468.81 (364.74-615.22)	11 (7-14)	3.61 (2.59-4.61)	372 (283-474)	88.58 (67.65-110.66)
Ethiopia	17579 (13804-22707)	42.11 (32.38-55.88)	184880 (146237-239761)	479.99 (377.48-630.3)	697 (360-1128)	2.52 (1.29-4.11)	25179 (17422-35300)	70.26 (48.29-98.85)
Fiji	428 (326-563)	61.36 (46.49-80.16)	4434 (3483-5800)	676.96 (527.93-884.27)	26 (20-33)	6.7 (5.37-8)	843 (664-1017)	144.64 (115.55-171.88)
Finland	8476 (6391-11009)	69.89 (55.04-86.68)	117234 (94965-141038)	862.54 (725.46-1013.76)	794 (645-877)	4.67 (3.87-5.13)	18394 (14929-21967)	127.35 (103.37-152.38)
France	78372 (57303-105675)	59.15 (44.34-78.68	1081926 (820574-1416350	717.98 (554.42-925.96	9758 (8012-10779)	4.53 (3.78-4.99)	182910 (150493-221951)	107.36 (86.51-132.2

location	Incidence		Prevalence		Deaths		DALYs	
	Number (95% UI)	ASIR (95% UI, per 100,000)	Number (95% UI)	ASPR (95% UI, per 100,000)	Number (95% UI)	ASMR (95% UI, per 100,000)	Number (95% UI)	ASDR (95% UI, per 100,000)
		)	)	)				9)
Gabon	407 (313-532)	42.53 (32.55-56.71)	4065 (3180-5323)	465.31 (360.35-610.14)	41 (31-50)	6.94 (5.29-8.37)	993 (774-1212)	129.32 (101.95-154.99)
Gambia	342 (265-451)	37.4 (28.57-50.28)	3491 (2752-4643)	411.42 (317.56-536.48)	29 (22-36)	5.11 (3.91-6.43)	680 (527-843)	93.4 (73.11-114.03)
Georgia	2830 (2114-3690)	47.21 (35.92-61.51)	35412 (26991-46236)	568.73 (436.23-738.62)	341 (293-380)	5.05 (4.37-5.62)	7760 (6545-9213)	122.39 (102.9-145.33)
Germany	140086 (122558-158105)	81.28 (72.34-89.74)	2155152 (1896612-2379617)	1072.86 (943.99-1172.82)	20539 (16509-22874)	7.81 (6.35-8.66)	397403 (335970-463533)	176.87 (148.23-208.48)
Ghana	6048 (4667-7938)	39.08 (29.56-51.95)	60669 (47244-80034)	432.42 (334.33-567.15)	346 (267-424)	4.26 (3.27-5.27)	9818 (7715-12324)	84.84 (67.39-103.92)

location	Incidence		Prevalence		Deaths		DALYs	
	Number (95% UI)	ASIR (95% UI, per 100,000)	Number (95% UI)	ASPR (95% UI, per 100,000)	Number (95% UI)	ASMR (95% UI, per 100,000)	Number (95% UI)	ASDR (95% UI, per 100,000)
Greece	12367 (9012-16893)	54.67 (41-71.66)	175318 (132665-230098)	662.08 (510.11-855.56)	1527 (1272-1673)	4.25 (3.6-4.62)	30788 (25091-37704)	105.1 (84.64-130.31)
Greenland	60 (44-79)	86.59 (65.04-114.49)	617 (478-795)	1014.83 (775.76-1305.09)	3 (2-4)	7.37 (5.55-9.35)	104 (81-130)	181.89 (144.48-226.12)
Grenada	65 (49-86)	58.42 (44.63-77.91)	695 (538-916)	653.72 (506.53-851.89)	5 (5-6)	7.08 (6.12-7.69)	135 (113-159)	141.52 (120.78-164.76)
Guam	126 (97-164)	59.14 (45.26-77.38)	1453 (1138-1871)	672.25 (525.78-866.79)	5 (4-6)	2.08 (1.64-2.41)	195 (152-244)	90.63 (70.16-113.59)
Guatemala	6076 (4623-8090)	56.47 (42.71-75.76)	66296 (51813-86475)	636.34 (499.2-825.44)	358 (309-404)	4.32 (3.74-4.84)	9969 (8055-12254)	102.34 (83.66-124.24)
Guinea	1867	35.62	19024	387.59	153	4.33	3582	82.81

location	Incidence		Prevalence		Deaths		DALYs	
	Number (95% UI)	ASIR (95% UI, per 100,000)	Number (95% UI)	ASPR (95% UI, per 100,000)	Number (95% UI)	ASMR (95% UI, per 100,000)	Number (95% UI)	ASDR (95% UI, per 100,000)
	(1435-2453)	(26.88-47.94)	(14744-25081)	(300.45-506.19)	(110-198)	(3.15-5.56)	(2749-4497)	(63.8-103.74)
Guinea-Bissau	233 (180-308)	36.54 (27.84-49.11)	2225 (1727-2948)	394.7 (304.39-521.63)	14 (11-19)	5.1 (3.79-6.68)	417 (314-533)	94.52 (72.48-119.8 6)
Guyana	364 (277-481)	59.63 (45.02-79.68)	3827 (2984-5099)	667.24 (519.54-874.3)	24 (20-29)	5.58 (4.58-6.68)	681 (543-834)	127.56 (103.37-152. 5)
Haiti	3950 (3038-5216)	59.42 (44.98-79.53)	39243 (30771-51649)	654.08 (507-860.17)	219 (147-326)	5.63 (3.8-8.29)	6840 (4956-9180)	127.46 (93.23-171.6 9)
Honduras	3592 (2759-4716)	58.92 (45.29-77.41)	37962 (29758-49433)	664.18 (516.7-857.85)	302 (230-372)	7.51 (5.71-9.15)	7556 (5799-9174)	148.88 (115.58-180. 09)
Hungary	8870 (6408-11720)	46.18 (34.66-60.55	118850 (90414-156204)	574.65 (443.45-748.72	796 (678-894)	3.55 (3.03-3.99)	19640 (15966-23931)	92.84 (75.06-114.3

location	Incidence		Prevalence		Deaths		DALYs	
	Number (95% UI)	ASIR (95% UI, per 100,000)	Number (95% UI)	ASPR (95% UI, per 100,000)	Number (95% UI)	ASMR (95% UI, per 100,000)	Number (95% UI)	ASDR (95% UI, per 100,000)
		)		)				3)
Iceland	375 (296-464)	65.91 (52.74-81.71)	4788 (3892-5821)	793.27 (651.04-960.17)	51 (40-58)	6.84 (5.49-7.68)	945 (793-1109)	144.46 (120.93-170.09)
India	570121 (430165-762853)	51.45 (38.36-68.54)	5612366 (4314558-7360330)	533.92 (412.71-700)	27888 (20426-34928)	3.44 (2.5-4.32)	855145 (645560-1094554)	86.05 (65.71-108.52)
Indonesia	145423 (112648-190123)	66.36 (50.84-87.95)	1474160 (1152713-1922564)	728.2 (569.74-942.29)	6987 (5445-8421)	6.36 (4.84-7.8)	223354 (178284-274232)	133.13 (106.77-160.61)
Iran (Islamic Republic of)	29418 (22665-38723)	40.56 (30-54.38)	294249 (230024-382166)	425.39 (327.17-559.23)	1949 (1463-2233)	3.31 (2.47-3.8)	47525 (37528-58271)	72.39 (57.36-88.32)
Iraq	8151 (6210-10703)	38.36 (28.13-51.24)	78701 (61203-102060)	406.12 (308.47-532.03)	647 (481-826)	4.85 (3.51-6.19)	16445 (13020-19938)	93.63 (74.24-114)

location	Incidence		Prevalence		Deaths		DALYs	
	Number (95% UI)	ASIR (95% UI, per 100,000)	Number (95% UI)	ASPR (95% UI, per 100,000)	Number (95% UI)	ASMR (95% UI, per 100,000)	Number (95% UI)	ASDR (95% UI, per 100,000)
Ireland	4292 (3141-5685)	54.42 (40.84-71.55)	54616 (42201-70424)	666.48 (516.95-858.36)	456 (371-510)	5.2 (4.23-5.8)	9551 (7848-11621)	113.62 (93.02-138.37)
Israel	11110 (8995-12779)	91.98 (75.23-105.16)	147352 (122034-167473)	1155.51 (958.33-1312.39)	714 (580-794)	4.84 (3.98-5.35)	19691 (15654-24214)	148.81 (117.35-184.36)
Italy	99307 (72177-134792)	70.54 (52.49-92.64)	1327156 (1001901-1743600)	821.57 (632.6-1065.6)	9825 (7691-11040)	4.52 (3.6-5.05)	206967 (163080-257741)	116.35 (91.14-146.4)
Jamaica	1851 (1423-2396)	59.1 (44.9-77.87)	21347 (16750-27574)	675.56 (527.03-877.87)	206 (164-251)	5.46 (4.36-6.68)	4111 (3349-4961)	123.13 (99.37-149.66)
Japan	107285 (77975-144807)	31.86 (24.84-41.91)	1498702 (1171946-1964169)	391.87 (310.63-511.22)	13837 (10679-15687)	2.33 (1.86-2.58)	270641 (220302-330702)	61.78 (50.37-76.42)
Jordan	2472	37	23983	396.61	103	2.72	3377	63.06

location	Incidence		Prevalence		Deaths		DALYs	
	Number (95% UI)	ASIR (95% UI, per 100,000)	Number (95% UI)	ASPR (95% UI, per 100,000)	Number (95% UI)	ASMR (95% UI, per 100,000)	Number (95% UI)	ASDR (95% UI, per 100,000)
	(1878-3282)	(27.48-49.67)	(18451-31112)	(300.63-520.3)	(80-125)	(2.12-3.29)	(2602-4223)	(49.36-78.25)
Kazakhstan	8829 (6626-11519)	48.85 (37.09-63.65)	96397 (74221-126067)	589.25 (453.42-768.59)	350 (305-395)	2.97 (2.57-3.34)	12990 (10082-16723)	85.35 (68.14-108.69)
Kenya	8973 (7020-11663)	42.21 (32.33-56.29)	90501 (71144-118517)	467.05 (366.94-610.02)	407 (264-581)	3.39 (2.18-4.95)	13715 (9944-18038)	80.37 (57.75-106.07)
Kiribati	35 (27-46)	55.96 (42.42-75.07)	347 (272-457)	598.52 (466.2-771.38)	1 (1-2)	3.86 (3.02-5.05)	56 (44-70)	102.38 (80.22-126)
Kuwait	1013 (804-1301)	37.87 (27.94-50.89)	9836 (7697-12638)	408.07 (310.58-530.22)	60 (48-72)	3 (2.4-3.57)	1525 (1212-1889)	66.57 (52.84-82.17)
Kyrgyzstan	1957 (1491-2572)	41.13 (31.43-53.83	20779 (16256-27052)	491.16 (377.25-639.22	73 (61-84)	2.17 (1.82-2.49)	2816 (2187-3622)	69.06 (54.42-88.3)

location	Incidence		Prevalence		Deaths		DALYs	
	Number (95% UI)	ASIR (95% UI, per 100,000)	Number (95% UI)	ASPR (95% UI, per 100,000)	Number (95% UI)	ASMR (95% UI, per 100,000)	Number (95% UI)	ASDR (95% UI, per 100,000)
		)		)				
Lao People's Democratic Republic	2333 (1816-3027)	54.97 (41.92-72.84)	23869 (18913-31406)	605.11 (475.86-782.28)	130 (102-169)	5.14 (4.04-6.72)	3842 (2968-4839)	111.23 (87.57-138.4 4)
Latvia	1701 (1429-1990)	47.4 (40.34-54.56)	25774 (22299-29649)	629.57 (547.41-715.02)	226 (194-250)	4.49 (3.88-4.98)	4759 (4088-5510)	107.11 (90.35-125.0 3)
Lebanon	2244 (1665-3001)	36.25 (26.97-48.31)	24591 (18725-32516)	387.14 (298.49-511.08)	239 (193-308)	3.41 (2.75-4.35)	4546 (3720-5509)	68.9 (56.31-84.18)
Lesotho	387 (293-514)	39.92 (30.15-53.63)	3809 (2967-5053)	423.73 (327.59-555.16)	22 (16-29)	3.53 (2.63-4.59)	699 (534-890)	83.87 (64.5-105.66)
Liberia	726 (570-941)	37.02 (27.98-49.14)	7117 (5638-9316)	408.89 (317.19-535.39)	50 (36-67)	4.52 (3.28-5.97)	1248 (940-1587)	85.7 (65.43-107.1 9)

location	Incidence		Prevalence		Deaths		DALYs	
	Number (95% UI)	ASIR (95% UI, per 100,000)	Number (95% UI)	ASPR (95% UI, per 100,000)	Number (95% UI)	ASMR (95% UI, per 100,000)	Number (95% UI)	ASDR (95% UI, per 100,000)
Libya	1777 (1361-2315)	37.69 (27.8-50.68)	17554 (13696-22623)	398.27 (304.76-518.53)	111 (69-163)	3.07 (1.9-4.49)	2950 (2127-3911)	70.05 (50.35-92.29)
Lithuania	2548 (1918-3354)	48.23 (36.45-62.39)	37996 (28984-48993)	634.2 (495.02-808.33)	328 (288-363)	4.52 (3.98-5.01)	6940 (5789-8373)	107.61 (88.1-131.18)
Luxembourg	681 (580-770)	65.48 (56.11-73.74)	8302 (7313-9305)	751.62 (663.73-841.08)	83 (70-92)	6.32 (5.38-7.03)	1569 (1347-1837)	133.18 (114.42-156.51)
Madagascar	3804 (2959-5017)	37.01 (28.34-48.94)	37729 (29479-49128)	417.24 (327.3-548.36)	271 (177-388)	5.12 (3.35-7.35)	8193 (5940-10725)	104.41 (74.57-139)
Malawi	2719 (2109-3516)	39.29 (30.1-52.01)	27955 (21964-36667)	445.44 (350.05-586.14)	122 (77-179)	2.94 (1.86-4.29)	4339 (3177-5754)	75.66 (55.07-100.82)
Malaysia	15962	58.73	174176	673.16	931	4.94	26502	112.56

location	Incidence		Prevalence		Deaths		DALYs	
	Number (95% UI)	ASIR (95% UI, per 100,000)	Number (95% UI)	ASPR (95% UI, per 100,000)	Number (95% UI)	ASMR (95% UI, per 100,000)	Number (95% UI)	ASDR (95% UI, per 100,000)
	(12194-20998)	(44.38-78.66)	(137798-228296)	(526.92-873.05)	(786-1069)	(4.12-5.74)	(20978-32395)	(91.2-135.64)
Maldives	181 (145-233)	54.1 (41.34-71.72)	1871 (1497-2409)	619.06 (486.08-803.07)	10 (7-12)	4.06 (2.99-5.05)	261 (204-337)	93.33 (73.33-118.42)
Mali	2815 (2156-3713)	35.17 (26.68-47.05)	28145 (22007-37160)	384.98 (298.11-507.1)	128 (90-170)	2.95 (2.12-3.84)	4155 (3145-5466)	66.21 (50.52-84.94)
Malta	512 (438-589)	51.15 (44.57-58)	6323 (5524-7076)	598.49 (529.84-673.49)	58 (47-66)	4.88 (3.97-5.49)	1180 (1005-1388)	107.08 (90.69-126.34)
Marshall Islands	16 (12-21)	52.48 (40.09-69.43)	157 (122-206)	578.23 (451.61-751.27)	1 (1-2)	7.35 (5.53-9.4)	41 (30-53)	156.76 (122.5-196.88)
Mauritania	767 (589-1011)	37.53 (28.39-50.3)	8034 (6250-10647)	419.11 (324.19-548.09	73 (54-96)	5.36 (4.02-7.01)	1595 (1232-1996)	95.91 (73.63-118.2

location	Incidence		Prevalence		Deaths		DALYs	
	Number (95% UI)	ASIR (95% UI, per 100,000)	Number (95% UI)	ASPR (95% UI, per 100,000)	Number (95% UI)	ASMR (95% UI, per 100,000)	Number (95% UI)	ASDR (95% UI, per 100,000)
				)				2)
Mauritius	1022 (779-1353)	56.83 (43.14-75.26)	11425 (8978-15014)	642.42 (500.8-837.33)	76 (66-82)	4.84 (4.24-5.26)	1849 (1526-2231)	109.11 (90.73-131.0 2)
Mexico	82432 (63607-109361)	66.4 (50.87-88.29)	899391 (705327-1167818)	746.46 (582.69-967.96)	5147 (4611-5659)	4.86 (4.33-5.33)	139470 (110869-170421)	119.77 (96.17-145.2 6)
Micronesia (Federated States of)	35 (27-46)	53.81 (41.12-71.15)	353 (275-461)	586.69 (458.47-759.65)	3 (2-4)	8.86 (6.48-11.4 4)	95 (74-118)	173.09 (134.52-216. 49)
Monaco	55 (40-74)	58.82 (44.19-76.65)	754 (573-971)	718.17 (557.76-916.81)	6 (4-7)	4.35 (3.29-5.49)	125 (97-156)	109.17 (84.56-138.6 7)
Mongolia	1034 (782-1341)	45.64 (34.27-60.09)	10601 (8189-13897)	544.58 (413.48-704.44)	26 (20-33)	1.91 (1.49-2.44)	1288 (954-1703)	69.41 (52.42-89.83)

location	Incidence		Prevalence		Deaths		DALYs	
	Number (95% UI)	ASIR (95% UI, per 100,000)	Number (95% UI)	ASPR (95% UI, per 100,000)	Number (95% UI)	ASMR (95% UI, per 100,000)	Number (95% UI)	ASDR (95% UI, per 100,000)
Montenegro	484 (353-637)	47.55 (35.29-62.08)	5917 (4506-7731)	597.77 (457.54-773.59)	133 (107-163)	17.26 (13.44-21.44)	2331 (1944-2768)	266.09 (221.18-320.05)
Morocco	11738 (8720-15856)	36.59 (26.88-49.55)	117079 (89916-151800)	383.34 (292.51-505.95)	991 (711-1280)	4.28 (3.05-5.49)	23355 (18287-29438)	83.58 (65.59-104.3)
Mozambique	3914 (3015-5091)	38.13 (28.99-50.32)	39677 (30931-51541)	427.96 (333.79-561.38)	252 (156-395)	4.34 (2.74-6.93)	7582 (5257-10573)	92.79 (65.44-130.27)
Myanmar	25401 (19548-33339)	55.75 (42.05-74.46)	267795 (211909-352206)	614.44 (480.43-795.77)	1630 (1236-2084)	5.15 (3.9-6.6)	43946 (34393-56178)	111.54 (88.01-140.63)
Namibia	515 (390-681)	40.81 (31.04-55.47)	5143 (3995-6875)	443.93 (340.35-581.29)	29 (21-40)	3.77 (2.68-5.09)	897 (677-1147)	84.98 (63.14-107.72)
Nauru	3	60.59	31	671.41	0	10.35	9	204.45

location	Incidence		Prevalence		Deaths		DALYs	
	Number (95% UI)	ASIR (95% UI, per 100,000)	Number (95% UI)	ASPR (95% UI, per 100,000)	Number (95% UI)	ASMR (95% UI, per 100,000)	Number (95% UI)	ASDR (95% UI, per 100,000)
	(2-4)	(45.99-80.04)	(25-40)	(528.4-873.97)	(0-0)	(6.38-18.44)	(7-11)	(146.73-299.14)
Nepal	9675 (7232-12862)	44.58 (33.43-59.27)	96213 (73203-125250)	471.74 (359.9-618.89)	589 (401-829)	4.05 (2.76-5.8)	16079 (11993-21120)	87.28 (65.75-116.28)
Netherlands	22455 (18709-27075)	64.02 (54.48-75.58)	287028 (243038-332612)	769.98 (654.22-894.08)	2501 (2084-2738)	6.01 (5.03-6.57)	50673 (42989-59870)	130.44 (110.01-154.87)
New Zealand	5817 (4707-7311)	69.78 (57.18-86.67)	72708 (60818-88247)	835.21 (704.87-1004.71)	729 (593-805)	7.52 (6.14-8.3)	14013 (11814-16401)	154.18 (130.39-181.27)
Nicaragua	2773 (2128-3642)	58.13 (44.01-77.39)	30250 (23880-39026)	661.52 (516.78-851.32)	137 (111-165)	3.7 (3-4.48)	4233 (3296-5278)	97.61 (76.83-120.84)
Niger	2620 (1999-3479)	35.28 (26.73-47.18	25954 (20174-34414)	383.57 (295.92-504.59	114 (57-174)	3.07 (1.61-4.71)	3721 (2518-5115)	66.3 (43.73-90.92

location	Incidence		Prevalence		Deaths		DALYs	
	Number (95% UI)	ASIR (95% UI, per 100,000)	Number (95% UI)	ASPR (95% UI, per 100,000)	Number (95% UI)	ASMR (95% UI, per 100,000)	Number (95% UI)	ASDR (95% UI, per 100,000)
		)		)				)
Nigeria	35723 (28008-46500)	42.52 (32.42-56.76)	353475 (278100-466330)	461.68 (358.97-602.32)	2185 (1644-2671)	4.48 (3.44-5.46)	56183 (42281-70658)	87.08 (65.99-108.4)
Niue	1 (1-2)	60.47 (46.2-79.8)	14 (11-19)	677.63 (531.4-878.21)	0 (0-0)	7.39 (5.57-8.79)	3 (3-4)	155.44 (124.6-184.55)
North Macedonia	1603 (1158-2145)	45.77 (33.88-60.43)	18793 (14283-24572)	572.89 (435.57-741.4)	149 (110-214)	7.45 (5.8-9.5)	3664 (2801-4933)	136.26 (107.56-174.16)
Northern Mariana Islands	28 (21-37)	58.21 (44.24-76.14)	294 (229-378)	662.43 (515.37-853.03)	2 (2-3)	7.7 (6.44-8.85)	64 (53-78)	158.63 (132.79-185.55)
Norway	6082 (4530-8012)	62.72 (47.43-82.17)	79412 (61345-102809)	761.48 (589.35-985.49)	778 (629-857)	5.94 (4.86-6.51)	14671 (12145-17639)	128.56 (105.7-156.78)

location	Incidence		Prevalence		Deaths		DALYs	
	Number (95% UI)	ASIR (95% UI, per 100,000)	Number (95% UI)	ASPR (95% UI, per 100,000)	Number (95% UI)	ASMR (95% UI, per 100,000)	Number (95% UI)	ASDR (95% UI, per 100,000)
Oman	643 (504-829)	34.43 (25.27-46.32)	5827 (4557-7637)	365.26 (276.39-482.95)	45 (36-55)	4.73 (3.62-5.93)	1237 (991-1518)	90.96 (74.65-109.22)
Pakistan	57874 (44289-75861)	53.85 (40.3-71.6)	548174 (427141-726637)	558.61 (429.5-735.69)	3115 (2311-4332)	4.58 (3.33-6.38)	90387 (69208-116432)	101.51 (79.65-131.68)
Palau	12 (9-16)	56.92 (43.46-75.08)	125 (98-163)	646.55 (506.74-842.07)	1 (0-1)	4.69 (3.77-5.59)	19 (15-25)	113.24 (92.02-140.01)
Palestine	749 (570-982)	33.18 (24.4-44.66)	7197 (5525-9405)	347.45 (265.23-453.98)	68 (55-82)	5.37 (4.31-6.66)	1522 (1249-1838)	89.76 (74.27-107.6)
Panama	2656 (2054-3532)	59.92 (46.23-80.07)	30751 (24204-39735)	691.05 (542.43-895.33)	221 (170-263)	4.59 (3.55-5.46)	5027 (3957-6123)	110.42 (86.72-134.83)
Papua New	2219	49.67	21815	543.71	108	3.89	4124	102.24

location	Incidence		Prevalence		Deaths		DALYs	
	Number (95% UI)	ASIR (95% UI, per 100,000)	Number (95% UI)	ASPR (95% UI, per 100,000)	Number (95% UI)	ASMR (95% UI, per 100,000)	Number (95% UI)	ASDR (95% UI, per 100,000)
Guinea	(1744-2890)	(38.29-65.85)	(17006-28137)	(426.86-705.94)	(67-154)	(2.43-5.52)	(2976-5545)	(75.12-137.62)
Paraguay	3697 (2878-4841)	63.72 (49.18-84.12)	43040 (33767-55877)	769.35 (602.53-992.61)	290 (218-360)	5.73 (4.31-7.1)	7067 (5557-8778)	130.91 (103.06-161.94)
Peru	19333 (15032-25246)	57.88 (44.6-76.58)	220883 (171496-283755)	668.37 (516.03-860.23)	1169 (896-1501)	3.47 (2.66-4.45)	32300 (24716-40452)	97.33 (74.34-122.44)
Philippines	45632 (35428-59598)	60.3 (45.81-80.23)	466916 (369507-610795)	653.73 (513.13-843.12)	2445 (2054-2912)	4.82 (4.02-5.81)	72144 (56995-88259)	110.96 (89.17-135.18)
Poland	50025 (36265-66677)	69.46 (51.87-90.62)	648195 (491254-846914)	866.31 (664.47-1117.94)	4021 (3480-4421)	4.96 (4.31-5.45)	99702 (80721-122377)	130.17 (104.84-160.59)
Portugal	14593 (11898-18076)	59.99 (49.76-72.76	192005 (161459-229225)	695.71 (591.04-820.14	1173 (969-1288)	3.46 (2.9-3.78)	28100 (23004-34125)	96.53 (78.19-118.2

location	Incidence		Prevalence		Deaths		DALYs	
	Number (95% UI)	ASIR (95% UI, per 100,000)	Number (95% UI)	ASPR (95% UI, per 100,000)	Number (95% UI)	ASMR (95% UI, per 100,000)	Number (95% UI)	ASDR (95% UI, per 100,000)
		)		)				3)
Puerto Rico	4460 (3243-5952)	61.38 (45.95-80.92)	56494 (43078-73735)	715.09 (552.46-927.13)	390 (313-452)	3.82 (3.11-4.43)	8769 (7003-10860)	104.23 (82.57-131.44)
Qatar	366 (283-474)	37.6 (27.56-50.65)	3005 (2251-3953)	402.59 (305.76-529.29)	10 (5-15)	4.06 (1.45-6.04)	408 (298-555)	78.49 (45.53-103.69)
Republic of Korea	42194 (34817-52133)	47.21 (39.34-57.49)	628474 (544533-739494)	675.14 (584.7-790.59)	3165 (2356-3785)	3.5 (2.6-4.17)	89265 (71029-110072)	96.31 (76.81-117.99)
Republic of Moldova	2822 (2114-3649)	47.44 (36.08-60.91)	37430 (28839-48105)	622.47 (480.68-796.46)	215 (189-238)	3.5 (3.09-3.88)	6007 (4883-7306)	99.18 (80.48-120.79)
Romania	12515 (10602-15109)	33.74 (29.07-40.1)	162826 (141283-191969)	412.59 (359.76-488)	1429 (1263-1586)	3.35 (2.97-3.72)	31380 (26640-36872)	77.45 (65.23-91.39)

location	Incidence		Prevalence		Deaths		DALYs	
	Number (95% UI)	ASIR (95% UI, per 100,000)	Number (95% UI)	ASPR (95% UI, per 100,000)	Number (95% UI)	ASMR (95% UI, per 100,000)	Number (95% UI)	ASDR (95% UI, per 100,000)
Russian Federation	125499 (94653-165367)	52.32 (40.22-68.2)	1639057 (1272601-2138452)	673.56 (526.09-872.26)	10825 (9597-11704)	4.44 (3.92-4.8)	271806 (222263-328016)	111.42 (91.12-134.81)
Rwanda	2248 (1751-2936)	37.96 (29.04-50)	23218 (18165-30751)	431.09 (337.04-563.82)	102 (42-179)	3.03 (1.24-5.39)	3524 (2244-5177)	73.71 (45.42-110.15)
Saint Kitts and Nevis	39 (29-51)	58.19 (44.27-77.42)	406 (319-530)	657.86 (511.85-859.19)	3 (3-3)	7.65 (6.77-8.39)	77 (63-92)	145.54 (123.59-168.74)
Saint Lucia	143 (109-187)	59.78 (45.19-77.74)	1618 (1260-2099)	680.63 (528.46-880.89)	16 (13-19)	7.2 (5.98-8.44)	316 (255-375)	137.1 (111.28-162.68)
Saint Vincent and the Grenadines	84 (64-112)	59.11 (44.99-78.29)	939 (732-1231)	670.59 (523.25-876.28)	8 (7-9)	7.43 (6.63-8.15)	182 (152-215)	141.74 (120.9-165.99)
Samoa	77	57.07	825	637.38	7	6.72	180	146.13

location	Incidence		Prevalence		Deaths		DALYs	
	Number (95% UI)	ASIR (95% UI, per 100,000)	Number (95% UI)	ASPR (95% UI, per 100,000)	Number (95% UI)	ASMR (95% UI, per 100,000)	Number (95% UI)	ASDR (95% UI, per 100,000)
	(60-102)	(43.2-75.97)	(649-1086)	(497.38-825.04)	(5-9)	(5.11-8.46)	(143-224)	(115.9-180.16)
San Marino	41 (30-54)	58.5 (43.3-76.78)	583 (442-761)	720.08 (555.9-936.44)	4 (2-5)	3.03 (2.03-4.31)	84 (64-111)	92.22 (69.55-122.85)
Sao Tome and Principe	41 (32-53)	38.74 (29.34-52.48)	413 (324-548)	431.62 (332.48-560.79)	3 (2-4)	4.94 (3.79-5.87)	74 (59-90)	91.18 (73.63-109.63)
Saudi Arabia	6096 (4757-7921)	36.92 (27.2-49.85)	54167 (42125-71479)	391.86 (301.89-511.74)	249 (197-305)	3.35 (2.67-4.11)	9049 (7157-11409)	72.24 (58.16-88.58)
Senegal	2744 (2111-3606)	37.78 (28.58-50.72)	28239 (22128-37630)	416.56 (324.04-545.14)	232 (173-291)	5.03 (3.76-6.29)	5350 (4182-6681)	92.09 (72.76-112.97)
Serbia	5688 (4597-7029)	34.05 (28.15-41.75	70792 (59259-87626)	409.9 (345.15-506.86	810 (665-962)	4.63 (3.83-5.5)	16205 (13504-19228)	92.99 (77.52-110.3

location	Incidence		Prevalence		Deaths		DALYs	
	Number (95% UI)	ASIR (95% UI, per 100,000)	Number (95% UI)	ASPR (95% UI, per 100,000)	Number (95% UI)	ASMR (95% UI, per 100,000)	Number (95% UI)	ASDR (95% UI, per 100,000)
		)		)				6)
Seychelles	64 (50-83)	57.24 (43.64-74.83)	696 (551-905)	644.8 (506.5-834.04)	5 (4-6)	5.46 (4.33-6.73)	113 (90-136)	114.22 (91.68-137.86)
Sierra Leone	1403 (1091-1862)	39.73 (30.33-53.31)	14273 (11176-18823)	438.39 (341.01-578.66)	82 (61-106)	3.86 (2.96-4.89)	2275 (1713-2918)	80.38 (61.47-101.1)
Singapore	2861 (2242-3726)	33.07 (25.69-42.97)	36795 (28982-47620)	430.26 (339.42-557.39)	99 (84-108)	1.22 (1.03-1.33)	4278 (3235-5584)	50.52 (38.39-65.59)
Slovakia	7231 (5730-8496)	73.15 (58.9-85.48)	90634 (74283-106522)	917.33 (758.01-1072.43)	562 (463-658)	5.99 (4.9-7.02)	14612 (11797-17705)	150.56 (121.36-182.31)
Slovenia	2024 (1731-2316)	48.42 (42.05-54.65)	28595 (25053-32065)	617.35 (543.74-691.9)	214 (175-242)	3.67 (3.01-4.15)	4654 (3899-5468)	92.57 (76.97-109.58)

location	Incidence		Prevalence		Deaths		DALYs	
	Number (95% UI)	ASIR (95% UI, per 100,000)	Number (95% UI)	ASPR (95% UI, per 100,000)	Number (95% UI)	ASMR (95% UI, per 100,000)	Number (95% UI)	ASDR (95% UI, per 100,000)
Solomon Islands	160 (124-212)	51.92 (39.63-69.56)	1594 (1258-2087)	568.32 (443.6-747.82)	9 (5-13)	4.92 (2.65-8.18)	306 (217-408)	115.15 (78.89-160.17)
Somalia	1951 (1522-2560)	35.55 (27.45-47.35)	18687 (14347-24314)	392.02 (308.37-516.94)	61 (26-111)	2.23 (1.02-3.98)	2767 (1735-4169)	63.59 (39.51-97.45)
South Africa	21323 (16354-28253)	48.94 (37.27-64.92)	217357 (169171-286785)	526.19 (408.32-689.91)	1208 (1035-1329)	4.04 (3.43-4.51)	34786 (28411-42506)	91.41 (75.11-110.29)
South Sudan	1351 (1062-1777)	37.43 (28.5-49.65)	13605 (10696-17831)	423.26 (332.01-554.83)	63 (31-99)	2.97 (1.47-4.74)	2148 (1433-2959)	73.44 (48.25-101.88)
Spain	64456 (56846-73040)	69.9 (62.22-78.58)	902888 (806291-1004786)	863.92 (780.25-960.23)	6767 (5396-7550)	4.54 (3.69-5.04)	140604 (117419-165622)	119.53 (98.17-143.01)
Sri Lanka	14672	55.75	163229	632.17	777	4.05	22556	95.58

location	Incidence		Prevalence		Deaths		DALYs	
	Number (95% UI)	ASIR (95% UI, per 100,000)	Number (95% UI)	ASPR (95% UI, per 100,000)	Number (95% UI)	ASMR (95% UI, per 100,000)	Number (95% UI)	ASDR (95% UI, per 100,000)
	(11154-19567)	(42.39-73.31)	(128662-213322)	(494.65-817.05)	(556-1029)	(2.89-5.35)	(16762-28901)	(71.97-120.5 7)
Sudan	6614 (4970-8739)	37.78 (27.49-50.99)	63916 (49242-83110)	394.5 (298.41-515.66)	394 (287-519)	3.24 (2.32-4.25)	10967 (8324-13689)	72.54 (56.08-90.42)
Suriname	361 (277-477)	58.05 (44.73-77.23)	3954 (3098-5171)	651.22 (508.16-840.34)	29 (21-37)	5.28 (3.89-6.83)	706 (544-876)	119.94 (93.43-148.7 5)
Sweden	25710 (18631-33708)	123.84 (92.51-159.6 7)	360875 (270489-465091)	1529.82 (1166.57-1943. 26)	2851 (2296-3193)	9.47 (7.71-10.5 9)	57180 (45730-70747)	222 (175.69-277. 17)
Switzerland	6346 (5485-7579)	36.45 (31.83-42.53)	84810 (74812-97781)	439.01 (388.91-505.6)	773 (609-867)	3 (2.39-3.34)	14918 (12437-17545)	69.34 (57.59-82.94)
Syrian Arab Republic	4228 (3163-5674)	35.28 (25.74-47.13	41606 (32220-54570)	377.81 (288.12-497.32	318 (210-419)	4.57 (2.89-5.92)	8108 (6087-10069)	85.65 (63.76-105.2

location	Incidence		Prevalence		Deaths		DALYs	
	Number (95% UI)	ASIR (95% UI, per 100,000)	Number (95% UI)	ASPR (95% UI, per 100,000)	Number (95% UI)	ASMR (95% UI, per 100,000)	Number (95% UI)	ASDR (95% UI, per 100,000)
		)		)				3)
Taiwan (Province of China)	22565 (17990-28132)	53.21 (42.42-67.12)	273226 (222314-341516)	629.83 (513.33-784.23)	1771 (1474-1977)	3.71 (3.12-4.12)	41012 (33444-50586)	92.14 (74.48-113.99)
Tajikistan	2333 (1786-3072)	40.85 (30.53-53.3)	23850 (18509-31221)	489.48 (373.73-641.48)	39 (32-48)	1.15 (0.92-1.44)	2547 (1826-3444)	54.26 (39.71-72.32)
Thailand	57918 (44292-77052)	53.17 (40.48-70.77)	661374 (516881-864805)	603.8 (470.51-784.54)	5064 (3744-6440)	4.56 (3.39-5.83)	110567 (87502-136071)	101.05 (80.12-124.39)
Timor-Leste	439 (332-586)	54.61 (41.79-72.26)	4680 (3685-6092)	605.83 (476.38-787.01)	22 (17-28)	4.54 (3.47-5.8)	692 (531-906)	102.36 (80.67-131.25)
Togo	1305 (1010-1717)	37.53 (28.56-49.83)	12756 (9931-16740)	411.66 (322.28-540.75)	77 (57-98)	4.7 (3.6-5.93)	2198 (1650-2761)	89.23 (69.02-110.26)

location	Incidence		Prevalence		Deaths		DALYs	
	Number (95% UI)	ASIR (95% UI, per 100,000)	Number (95% UI)	ASPR (95% UI, per 100,000)	Number (95% UI)	ASMR (95% UI, per 100,000)	Number (95% UI)	ASDR (95% UI, per 100,000)
Tokelau	1 (1-1)	56.13 (42.64-73.71)	9 (7-12)	629.56 (494.07-812.57)	0 (0-0)	7.16 (4.95-10.39)	2 (2-3)	149.97 (112.24-200.51)
Tonga	46 (35-60)	58.48 (44.53-76.74)	507 (400-655)	654.77 (516.14-842.08)	4 (3-5)	5.01 (3.51-6.87)	91 (71-117)	119.97 (93.23-153.34)
Trinidad and Tobago	1240 (932-1641)	63.93 (48.81-84.75)	14037 (10919-18399)	732.58 (567.98-954.23)	99 (77-119)	5.64 (4.41-6.79)	2386 (1898-2915)	128.6 (102.59-156.53)
Tunisia	4414 (3251-5838)	34.48 (25.02-45.65)	45069 (34412-58372)	365.01 (276.78-471.64)	463 (305-649)	4.61 (2.98-6.69)	9283 (6895-12247)	81.82 (60.46-107.55)
Turkey	24774 (21786-28278)	27.35 (24.06-31.31)	247837 (221168-275262)	282.89 (252.21-314.68)	2923 (2332-3533)	3.88 (3.05-4.69)	55942 (46418-65880)	67.92 (56.42-80.43)
Turkmenistan	1872	47.04	19896	563.75	86	2.89	2975	86.5

location	Incidence		Prevalence		Deaths		DALYs	
	Number (95% UI)	ASIR (95% UI, per 100,000)	Number (95% UI)	ASPR (95% UI, per 100,000)	Number (95% UI)	ASMR (95% UI, per 100,000)	Number (95% UI)	ASDR (95% UI, per 100,000)
	(1439-2433)	(35.47-60.99)	(15619-25583)	(432.79-729.21)	(68-112)	(2.27-3.76)	(2299-3738)	(67.67-106.69)
Tuvalu	5 (4-7)	55.34 (42.18-72.65)	57 (45-75)	609.07 (478.07-796.75)	0 (0-1)	7.14 (5.55-8.84)	13 (10-16)	150.94 (121.58-183.48)
Uganda	5416 (4239-7160)	38.93 (29.92-52.22)	55891 (43879-73062)	442.48 (347.59-586.14)	251 (126-429)	2.91 (1.43-4.96)	8476 (5794-12027)	73.52 (49.36-105.55)
Ukraine	35530 (26656-46895)	46.19 (35.34-60.16)	468750 (362555-614415)	586.74 (457.73-760.23)	3236 (2489-4051)	4.1 (3.16-5.13)	78770 (61400-97874)	98.56 (76.69-121.96)
United Arab Emirates	1662 (1236-2173)	39.65 (29.19-52.8)	13475 (9821-18266)	424.69 (326.48-554.82)	30 (24-37)	3.82 (2.51-4.93)	1763 (1273-2342)	85.82 (66.1-105.16)
United Kingdom	65549 (51519-83607)	52.25 (41.8-65.6)	888645 (725229-1093517	648.32 (535.59-793.81	9412 (7868-10176)	5.71 (4.8-6.15)	176779 (150586-207501)	119.73 (101.41-141.

location	Incidence		Prevalence		Deaths		DALYs	
	Number (95% UI)	ASIR (95% UI, per 100,000)	Number (95% UI)	ASPR (95% UI, per 100,000)	Number (95% UI)	ASMR (95% UI, per 100,000)	Number (95% UI)	ASDR (95% UI, per 100,000)
			)	)				22)
United Republic of Tanzania	9526 (7370-12404)	39.1 (30.07-51.91)	99864 (78447-132449)	445.22 (349.86-586.08)	580 (329-891)	3.6 (2.05-5.39)	16779 (11752-23254)	82.33 (57.78-113.5 3)
United States Virgin Islands	115 (83-157)	62.03 (47.02-82.25)	1367 (1038-1806)	710.98 (550.75-926.06)	8 (6-10)	5.15 (3.97-6.7)	201 (151-252)	113.8 (87.47-142)
United States of America	528208 (487191-57474 3)	89.18 (82.53-96.66)	6373868 (5938751-685294 4)	1040.36 (973.52-1116.2 5)	35285 (28993-38677)	5.26 (4.36-5.74)	916645 (768611-108029 4)	146.72 (122.77-172. 78)
Uruguay	2129 (1563-2898)	38.49 (28.98-51.8)	26364 (20167-34763)	445.88 (345.48-579.21)	277 (235-301)	3.78 (3.24-4.1)	5256 (4397-6336)	82.04 (68.63-99.71)
Uzbekistan	10746 (8148-14124)	41.51 (30.94-54.26)	112675 (87824-148398)	496.56 (380.6-651.61)	240 (204-278)	1.39 (1.18-1.6)	12966 (9657-17427)	58.96 (44.73-77.9)

location	Incidence		Prevalence		Deaths		DALYs	
	Number (95% UI)	ASIR (95% UI, per 100,000)	Number (95% UI)	ASPR (95% UI, per 100,000)	Number (95% UI)	ASMR (95% UI, per 100,000)	Number (95% UI)	ASDR (95% UI, per 100,000)
Vanuatu	93 (72-121)	59.69 (45.68-78.54)	942 (739-1229)	658.57 (516.53-852.91)	5 (3-6)	5.14 (3.8-6.54)	175 (135-222)	127.94 (98.97-160.53)
Venezuela (Bolivarian Republic of)	18248 (13775-24049)	61.32 (46.53-81.59)	202304 (158201-263377)	700.83 (544.82-906.43)	1204 (948-1481)	4.58 (3.63-5.6)	31981 (25013-39311)	114.29 (89.6-140.04)
Viet Nam	53983 (41974-71154)	57.26 (43.75-76.67)	581496 (457784-771131)	644.9 (502.66-844.07)	4015 (2990-5030)	5.83 (4.35-7.36)	97149 (74777-122074)	118.87 (91.48-147.64)
Yemen	4105 (3061-5427)	32.9 (24.21-44.14)	38432 (29592-49671)	337.23 (255.75-443.55)	291 (198-404)	3.74 (2.57-5.12)	7685 (5625-9928)	75.17 (55.62-97.35)
Zambia	2320 (1828-3002)	35.81 (27.43-47.89)	23356 (18374-30162)	404.89 (320.47-528.63)	218 (129-366)	5.71 (3.59-9.09)	5743 (3778-9002)	112.52 (76.94-168.22)
Zimbabwe	2534	41.28	24452	444.98	118	3.39	4171	82.48

location	Incidence		Prevalence		Deaths		DALYs	
	Number (95% UI)	ASIR (95% UI, per 100,000)	Number (95% UI)	ASPR (95% UI, per 100,000)	Number (95% UI)	ASMR (95% UI, per 100,000)	Number (95% UI)	ASDR (95% UI, per 100,000)
	(1926-3337)	(31.18-55.1)	(18907-32514)	(344.65-580.89)	(80-149)	(2.23-4.25)	(3086-5215)	(61.95-102)

Note: ASIR, age-standardized incidence rate; ASPR, age-standardized prevalence rate; ASMR, age-standardized mortality rate; ASDR, age-standardized DALYs rate; UI, uncertainty intervals.
